# The Role of Ion Size and π‐Interaction in Stabilizing Calix[4]arene Crown Ether Metal Complexes

**DOI:** 10.1002/chem.202501065

**Published:** 2025-05-21

**Authors:** Thomas Sittel, Karolin Becker, Robert Polly, Udo Müllich, Andreas Geist, Petra J. Panak

**Affiliations:** ^1^ Karlsruhe Institute of Technology Institute for Nuclear Waste Disposal P.O. Box 3640 76021 Karlsruhe Germany; ^2^ Institute for Physical Chemistry Heidelberg University Im Neuenheimer Feld 253 69120 Heidelberg Germany

**Keywords:** calixarenes, computational chemistry, main group elements, NMR spectroscopy, supramolecular chemistry

## Abstract

This study systematically investigates the influence of ion size on the structure and stability of complexes formed between the calix[4]arene crown ether 1,3‐alt‐25,27‐bis(3,7‐ dimethyloctyl‐1‐oxy)calix[4]arenebenzocrown‐6 (MAXCalix) and mono‐ and divalent ions from the alkali and alkaline earth metal series. NMR spectroscopy studies revealed that while MAXCalix efficiently coordinates large ions such as Cs^+^, it also forms complexes with smaller ions like Na^+^, highlighting the ligand's versatility. The size of the ion directly influences the complex structure, with two distinct structural subtypes identified via NMR and DFT calculations. In addition, π‐interactions between the cation and the cation‐facing benzene rings of the calix[4]arene backbone play an important role in stabilizing the complex. Larger ions like Cs^+^ benefit from π‐interactions with both cation‐facing rings, whereas smaller ions like K^+^ interact with only one ring, if any. These π‐interactions are primarily drivers of the enhanced affinity for Cs^+^ and the resulting higher complex stability. Competitive NMR studies further confirmed that the complex stability increases with increasing ionic radius, and ions of similar size show comparable stability of their complexes.

## Graphical abstract

This work investigates MAXCalix's ability to coordinate alkali and alkaline earth metal ions, showing a preference for larger ions like Cs⁺. Structural analysis confirms that π‐interactions with benzene rings enhance stability, particularly for large cations that engage both rings. Competitive NMR experiments validate that stability increases with ionic radius, demonstrating MAXCalix's strong affinity for cesium.

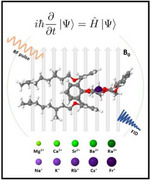

## 1 Introduction

**Scheme 1:**
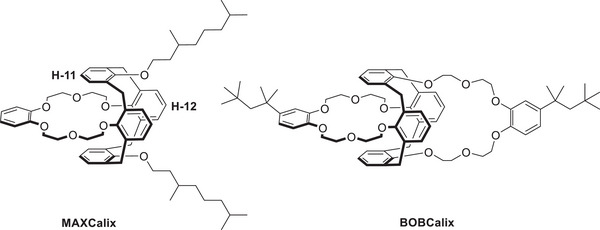
Molecule structures of the calix[4]arene 18‐crown‐6 ethers MAXCalix and BOBCalix.

Calixarenes and crown ethers are well‐known for their highly specific binding capabilities toward cations. Since the early 1990, hybrid compounds that integrate both structural motifs have been synthesized and studied extensively.^[^ ^1^, ^2^, ^3^, ^4^, ^5^, ^6^, ^7^, ^8^ ^]^ These hybrid structures can be tailored to selectively bind specific ions based on their size, making them particularly valuable for practical applications, such as extracting agents in nuclear waste processing.^[^ ^9^, ^10^, ^11^, ^12^, ^13^, ^14^ ^]^ Among the most promising agents are the calix[4]arene 18‐crown‐6 ethers calix[4]arene‐bis(t‐octylbenzo‐crown‐6) BOBCalix and 1,3‐alt‐25,27‐bis(3,7‐ dimethyloctyl‐1‐oxy)calix[4]arenebenzocrown‐6 (MAXCalix, structures shown in Scheme 1), which have demonstrated high efficiency in Cs^+^ extraction.^[^ ^15^, ^16^, ^17^, ^18^, ^19^, ^20^ ^]^ In recent years, various research groups have focused on revealing the molecular structure of Cs^+^ complexes with calix[4]arene crown ethers to better understand their extraction mechanisms using solvent extraction, NMR spectroscopy, x‐ray diffraction, and density functional theory.^[^ ^21^, ^22^, ^23^, ^24^, ^25^, ^26^, ^27^, ^28^ ^]^ Both, crystallographic data and DFT‐optimized structures of cesium complexes revealed significant π‐interactions between the metal‐facing aromatic rings of the calixarene and the Cs^+^ cation.

Our recent studies on the separation of Cs^+^ from monovalent and divalent cations have shown that Cs^+^ is extracted more efficiently than K^+^.^[^ ^29^ ^]^ At first glance, this may seem counterintuitive given the well‐established 18‐crown‐6 ether chemistry,^[^ ^30^, ^31^ ^]^ but highlights a gap in understanding the coordination chemistry of these ligands with chemically related ions. This underlines the need for a systematic approach to comprehensively study these interactions.

Motivated by the above‐mentioned results, we have investigated the complexation of alkali and alkaline earth metal ions with the calix[4]arene 18‐crown‐6 ether MAXCalix. NMR spectroscopy was employed to experimentally determine the complex structures and speciation for Na^+^, K^+^, NH_4_^+^, Rb^+^, Cs^+^, Mg^2+^, Ca^2+^, and Sr^2+^. Competitive NMR studies were conducted to establish relative complex stabilities in solution, verifying the results obtained from solvent extraction experiments. Complementary DFT calculations of the complex series further provide insights into the molecular structure, clarifying the underlying reasons for differences in complex stability.

## 2 Results and Discussion

### 2.1 Single‐Metal Ion Speciation

**Figure 1:**
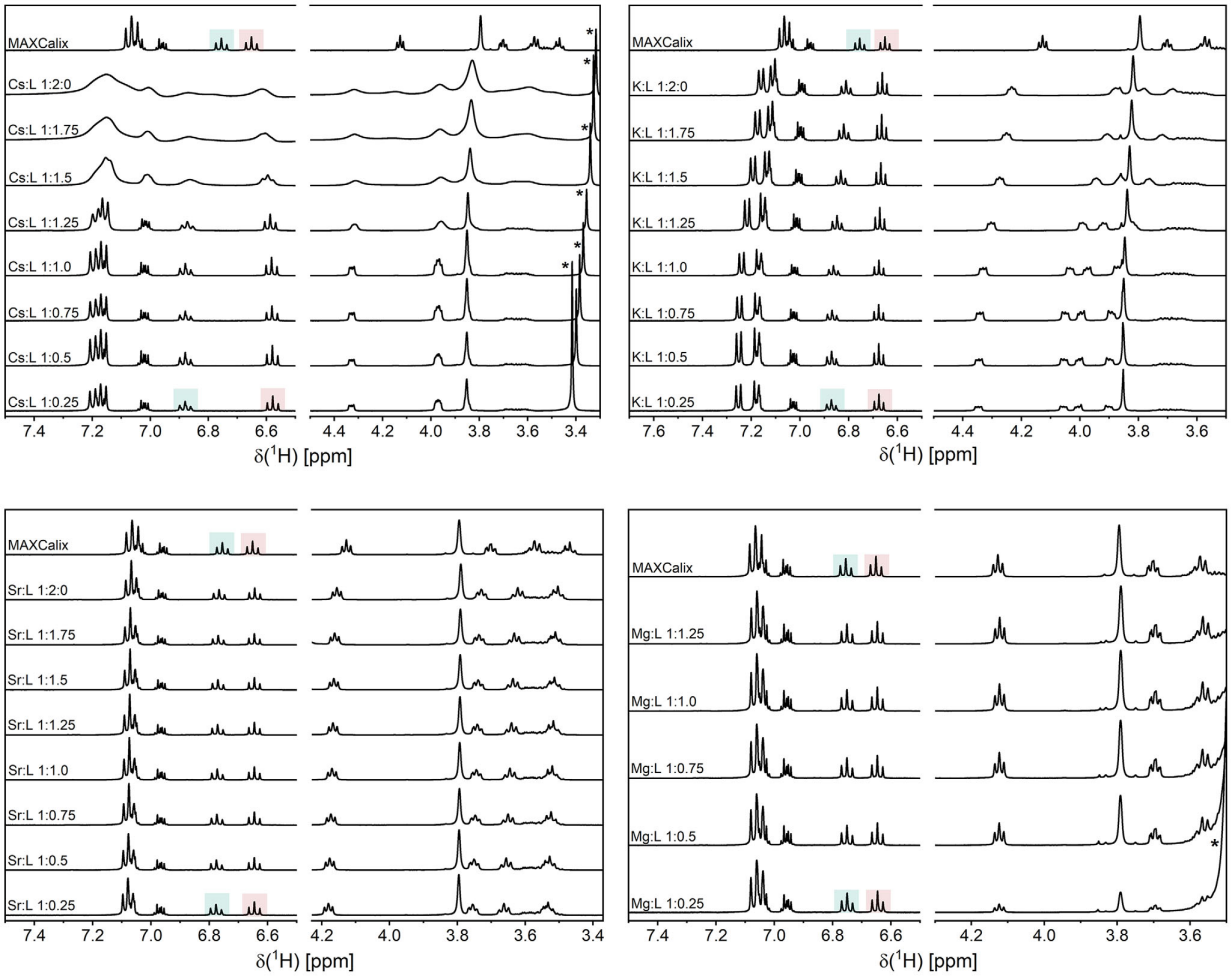
^1^H‐NMR spectra (400.13 MHz, 300 K) of the aromatic and crown ether region of MAXCalix depending on the MAXCalix/metal ion ratio for Cs^+^, K^+^, Sr^2+^, and Mg^2+^ ([M] = 1.7 × 10^−2^ mol L^−1^) in acetone‐d_6_. ■ H‐11, ■ H‐12, * δ(H_2_O).

MAXCalix has demonstrated its effectiveness as an extractant for cesium in various processes.^[^ ^16^, ^19^, ^20^, ^29^ ^]^ However, there is a surprising lack of complexation studies involving this ligand. To address this, we conducted speciation studies with MAXCalix using M^+^ ion (Na, K, Rb, Cs, NH_4_) and M^2+^ ions (Ca, Mg, Sr). Figure 1 provides an overview of the complexation progress for Cs^+^, K^+^ Mg^2+^, and Sr^2+^ with increasing M:L ratio (see Figure S1 for Rb^+^, NH_4_^+^, Na^+^, Ca^2+^). For Cs^+^, the formation of complexes significantly affects the chemical shifts in both the aromatic and crown ether region of the proton NMR spectra. The spectra remain consistent until the Cs:L ratio exceeds 1:1.00, when line broadening is observed. The observed line broadening is indicative of a rapid exchange between free and complexed ligand. This effect may also be attributed to aggregation processes in solution. Overall, we conclude that only a single complex species, [Cs(MAXCalix)]^+^, is formed. This conclusion is consistent with the ligand's structure, supported by available extraction data, and confirmed by ESI^+^ MS (found: 1087.5058, calculated: 1087.5064 for CsC_62_H_82_O_8_). In the aromatic region, most signals shift toward lower fields, except for the triplet assigned to H‐11. Consequently, the chemical shift difference between the aromatic protons H‐11 and H‐12 is 0.29 ppm. This difference in chemical shift of H‐11 and H‐12 was observed in NMR titration experiments with BOBCalix and Cs^+^, where the Cs:BOBCalix ratio remained below 1:1.^[^ ^25^ ^]^ This suggests a significant change in electronic density distribution between the metal‐facing and the metal‐opposing rings of the 1,3‐alternating calix[4]arene, indicating possible π‐interactions between the metal‐facing benzene rings and the cation. This trend is also reflected in the carbon NMR data, which show substantial differences in chemical shifts between the arene rings (see Figure S4). In the crown ether region, the CH_2_ groups of the crown ether appear at 4.33, 3.97 (2×), and 3.85 ppm. The significant changes in signal multiplicity compared to the free ligand indicate a conformational change in the crown ether substructure. Carbon NMR data further reveal that the chemical shifts for the CH_2_ groups closer to the calix[4]arene backbone experience greater shifts than those closer to the terminal benzene ring. This suggests that the cation is not symmetrically located within the crown ether but is displaced closer to the calix[4]arene rings, supporting the evidence for π‐interactions. In addition, the residual water signal is significantly shifted (*δ* = 3.39 ppm at Cs:L = 1:1.00) compared to the anticipated value in acetone‐d_6_ (2.95 ppm^[^ ^32^ ^]^). This shift indicates that residual water molecules are occupying the remaining coordination sites of the cesium cation. To investigate the fate of the triflate ion in solution, ^19^F NMR spectra were recorded (see Figure S5). The spectra show constant shifts at Cs:L ratios below 1:1.00, indicating that the anion is progressively separated from the cation during complex formation. At Cs:L exceeding 1:1.00, the ^19^F signal no longer shifts, with a chemical shift close to that of solvated triflate. This suggests that the complex and anion form a fully solvated ion pair for charge compensation, with no evidence of anion complexation.

For K^+^, the progression is similar to that observed for Cs^+^, with no noticeable changes until K:L exceeds 1:1.00. At that point, signal shifts indicate a rapid exchange or molecule aggregation. This suggests the formation of the [K(MAXCalix)]^+^ complex, which has been confirmed via ESI^+^ MS (found: 993.5640, calculated: 993.5646 for KC_62_H_82_O_8_). In the aromatic region, the difference between the two triplets is 0.19 ppm. In addition, the remaining signals of the calix[4]arene rings are more distinctly separated compared to those of the Cs^+^ complex or the unbound ligand. In the crown ether region, the four signals corresponding to the individual CH_2_ groups are also well resolved. The signal multiplicity is similar to that observed for the Cs^+^ complex, indicating a similar crown ether conformation. Unlike Cs^+^, the residual water signal is consistently found at 2.96 ppm throughout the titration series, indicating a closed‐shell K^+^ coordination sphere within the complex. Rb^+^ and NH_4_^+^ show similar NMR spectral features as Cs^+^ and K^+^ (see Figures S7, S11). Notably, in the NH_4_^+^ titration series, the NH_4_^+^ proton signal appears as a triplet due to coupling with ^14^N. Initially absent, this signal evolves from a broad peak at 5.1 ppm (NH_4_^+^:L ratio = 1:0.75) to a distinct triplet at 4.51 ppm as the ligand concentration and complex formation progresses. This indicates that the typically rapid exchange of NH_4_^+^ protons is significantly reduced when the cation is complexed.

In contrast, the complexation of Sr^2+^ is marked by relatively small shifts in the aromatic region. The difference in chemical shift between H‐11 and H‐12 is 0.14 ppm, compared to 0.10 ppm in the free ligand. The complexation is more evident in the crown ether region, where the triplets shift to lower field strengths relative to the free ligand, as observed in both proton and carbon NMR spectra (ESI^+^ MS: found: 1191.4593, calculated: 1191.4596 for SrC_62_H_82_O_8_CF_3_SO_3_). Unlike Cs^+^ and K^+^, the crown ether signals retain their triplet multiplicity, suggesting a different conformation of the crown ether in the Sr^2+^ complex. In addition, the residual water signal shifts from 4.50 to 4.25 ppm, indicating additional water coordination. Na^+^ shows a similar behavior, with complexation primarily reflected by subtle changes in the crown ether region (ESI^+^ MS: found: 977.5903, calculated: 977.5907 for NaC62H82O8). However, the data suggests only minimal complex formation, likely explaining the slight NMR shifts with increasing ligand concentration, indicative of an exchange mechanism between the unbound and complexed ligand.

For Mg^2+^, no significant changes were observed during the titration series compared to the spectrum of the unbound ligand, indicating that Mg^2+^ is not coordinated by the ligand, likely due to its small ionic size. This observation is consistent with results obtained in extraction experiments.^[^ ^29^ ^]^ The same applies to Ca^2+^, as no evidence of complex formation was observed in the NMR spectra or ESI^+^ MS data.

In summary, the formation of a 1:1 complex is evident for Cs^+^, Rb^+^, K^+^, NH_4_^+^, Na^+^, and Sr^2+^. There is a clear correlation between ionic size and complexation behavior. Cs^+^, Rb^+^, K^+^, and NH_4_^+^ show similar complex structures. The cation seemingly interacts with the arene rings via π‐interactions. In contrast, Sr^2+^ shows a different crown ether conformation, likely due to its smaller ionic radius and higher nuclear charge. For Na^+^, the NMR data suggest a complex structure similar to Sr^2+^, but the rapid exchange/aggregation observed hints that its complex structure may be more akin to its group counterparts.

### 2.2 Competitive Speciation Study

While these results provide substantial information on the complex structure and the interaction mechanism between the cation and the ligand, it does not explain the ligand's higher affinity toward Cs^+^ over the remaining ions. From lanthanide/actinide separation studies, it is known that the separation factor correlates with the difference in complex stability of the respective species.^[^ ^33^ ^]^ Following this logic, Cs^+^ forms complexes that are two orders of magnitude more stable than those formed by K^+^ (separation factor for Cs/K ∼ 100).^[^ ^29^ ^]^ These findings contradict expectations based solely on 18‐crown‐ether‐6 complexation chemistry, where K^+^ should form the strongest complex, and 21‐crown‐ether‐7 would be the ideal ligand for Cs^+^.^[^ ^30^, ^31^, ^34^ ^]^ To further investigate the molecular basis for this selectivity, we aim to determine the complex stability constants. However, to determination of stability constants for alkali and alkaline earth ions is not possible using NMR‐spectroscopy. Nevertheless, NMR is well‐suited for revealing relative trends within isostructural complexes. To address this, we have developed a simple procedure to study competitive complexation, which is detailed in the Supporting Information. This three‐step process is designed to identify which cation forms the strongest complex. The primary objective of this study is to establish a relative ranking of complex stability for [M(MAXCalix)]^n+^ with M = Cs^+^, K^+^, Na^+^, NH_4_^+^, Rb^+^, and Sr^2+^.

**Figure 2:**
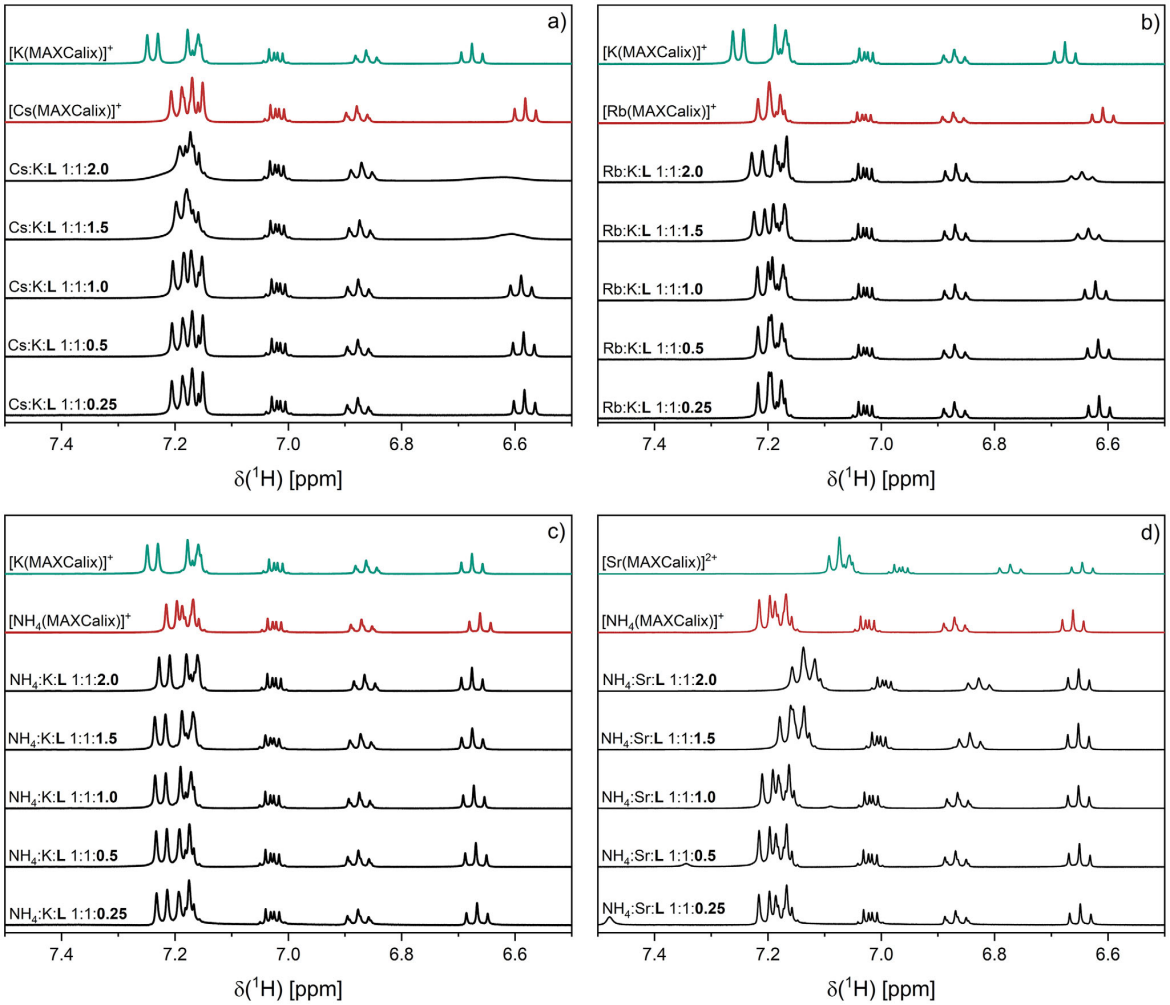
Evolution of the ^1^H‐NMR spectra (400.13 MHz, 300 K) of the aromatic region of MAXCalix depending on the MAXCalix/metal ion ratio for the ion pairs a) Cs^+^/K^+^, b) Rb^+^/K^+^, c) NH_4_^+^/K^+^, d) NH_4_^+^/Sr^2+^ ([M] = 1.7 · 10^−2^ mol L^−1^) in acetone‐d_6_.

Figure 2a shows the formation of [Cs(MAXCalix)]^+^ at Cs:K:L ratios ranging from 1:1:0.25 to 1:1:1.00. The resulting chemical shifts and signal patterns match with the data obtained in the single‐metal ion speciation, confirming that Cs^+^ is preferentially complexed by the ligand. Upon adding more ligand, the spectra change significantly between Cs:K:L ratios of 1:1:1.25 and 1:1:2.00. However, the expected formation of [K(MAXCalix)]^+^ is not directly observed, as the characteristic signals for the respective complex cannot be distinguished due to the overlap of signals from both complexes. With excess ligand, at Cs:K:L greater than 1:1:2.00, we observe significant line broadening, indicating rapid exchange between the coordinated and unbound ligand. As described earlier, Figure 2a only partially illustrates the steps taken to determine whether Cs^+^ or K^+^ forms the stronger complex. Additional data (see Figure S17) show that Cs^+^ can replace K^+^ from a pre‐formed K^+^ complex at low Cs^+^ equivalents, whereas K^+^ cannot replace Cs^+^ under similar conditions. Comparing Cs^+^ to Rb^+^, NH_4_^+^, or Na^+^ consistently shows Cs^+^ as the dominant complexing ion (see Figure S18).

Figure 2b shows the progression of the titration series for the Rb^+^/K^+^ ion pair. Between Rb:K:L ratios of 1:1:0.25 and 1:1:1.00, the Rb^+^ complex predominates. At a ratio of 1:1:1.00, slight changes are observed in the triplet at 7.2 ppm. With the addition of more ligand, the characteristic signals of the K^+^ complex at 7.18 and 7.22 ppm are clearly observable. The remaining signals show only minor changes, likely due to the similarity of the respective proton shifts in the K^+^ and Rb^+^ complexes. Overall, Rb^+^ is preferentially coordinated by the ligand over K^+^.

These competitive series suggest that complex stability is influenced by ionic size. K^+^ and NH_4_^+^ have similar ionic radii, so their complex stability should be comparable. Figure 2c shows the evolution of the proton spectrum with increasing ligand equivalents for the K^+^/NH_4_^+^ ion pair. At the beginning of the titration series, neither the characteristic signals of the K^+^ complex nor those of the NH_4_^+^ complex are observed. Instead, the pattern of the proton signal at 7.2 ppm suggests the simultaneous formation of both complexes. The addition of more ligand does not significantly affect the proton NMR, indicating that K^+^ and NH_4_^+^ form complexes of similar stability. This is confirmed by the additional titration series (see Figure S17).

Figure 2d shows the titration series for the NH_4_^+^/Sr^2+^ ion pair. At NH_4_^+^:Sr^2+^:L ratios below 1:1:1.00, only the ammonium complex is formed, with signals at 7.2 ppm identical to those observed in the single‐metal ion spectra. As the ligand concentration increases, the signals shift, indicating the formation of the Sr^2+^ complex. Similar to the Cs^+^/K^+^ pair, increasing NH_4_^+^ concentration results in the replacement of Sr^2+^ in the complex, while Sr^2+^ is unable to displace NH_4_^+^ in a pre‐formed [NH_4_(MAXCalix)]^+^ complex.

Based on the results discussed, we propose the following complex stability ranking for [M(MAXCalix)]^n+^

$${\mathrm{Cs}}^{+}>{\mathrm{Rb}}^{+}>{{\mathrm{NH}}_{4}}^{+}\approx K^{+}\gg {\mathrm{Sr}}^{2+}>{\mathrm{Na}}^{+}$$

As mentioned earlier, Cs^+^ forms the strongest complex with MAXCalix among the studied cations. High complex stability is a crucial requirement for developing an effective and selective extraction procedure. Following Cs^+^, Rb^+^ forms stronger complexes than NH_4_^+^ and K^+^. Sr^2+^ and Na^+^ form the weakest complexes, with Na^+^ forming an even weaker complex than Sr^2+^ in direct comparison. Although this stability ranking technically applies only to acetone, the correlation between complex stability and ion size is consistent with trends observed in recent extraction studies involving the separation of radioactive cesium from chloride‐rich brine solutions.^[^ ^29^ ^]^ Using a diluent mixture of 1‐octanol and kerosene, these studies report high separation factors for cesium over potassium and sodium, ranging from 100 to 1000. These separation factors reflect differences in complex stability and are in excellent agreement with the findings of the present spectroscopic study. Thus, the proposed stability ranking may also be extended to solvents that are more suitable for practical applications. In addition, NMR results suggest that this trend is due to a stronger interaction between the calix[4]arene aromatic rings and the larger cations, through π‐interactions. This stabilizing effect is evident in the proton and carbon NMR spectra of the complexes, as the difference in chemical shifts of H‐11/C‐11 and H‐12/C‐12 varies with ion size.

### 2.3 Complex Structure Study Using Density Functional Theory

**Figure 3:**
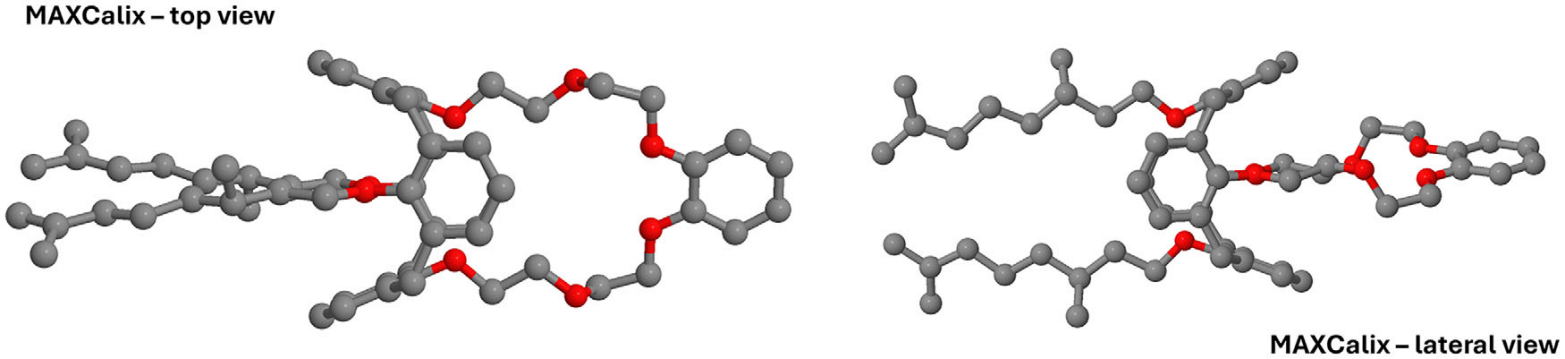
Top and lateral view of the DFT‐optimized structure of MAXCalix using BP86 functional with the def2‐TZVPP basis set. Hydrogen atoms are omitted for visual clarity.

To complement the NMR analysis, DFT calculations were performed to gain a deeper understanding of the complex structures. Figure 3 presents the optimized structure with C2 symmetry of the isolated MAXCalix using BP86 functional with the def2‐TZVPP basis set. In this structure, the oxygen atoms form a flat plane within the 18‐crown‐ether‐6, which is facilitated by the distinct spatial orientation of the O─C─C─O substructures. The planar structure is further extended by the terminal benzene ring. The calix[4]arene backbone adopts a 1,3‐alternate conformation, with distances of 739.2 and 730.1 pm between the opposing benzene rings, emphasizing the overall symmetry of the coordination site within the ligand. To minimize repulsive forces, the alkyl groups are slightly twisted.

**Figure 4:**
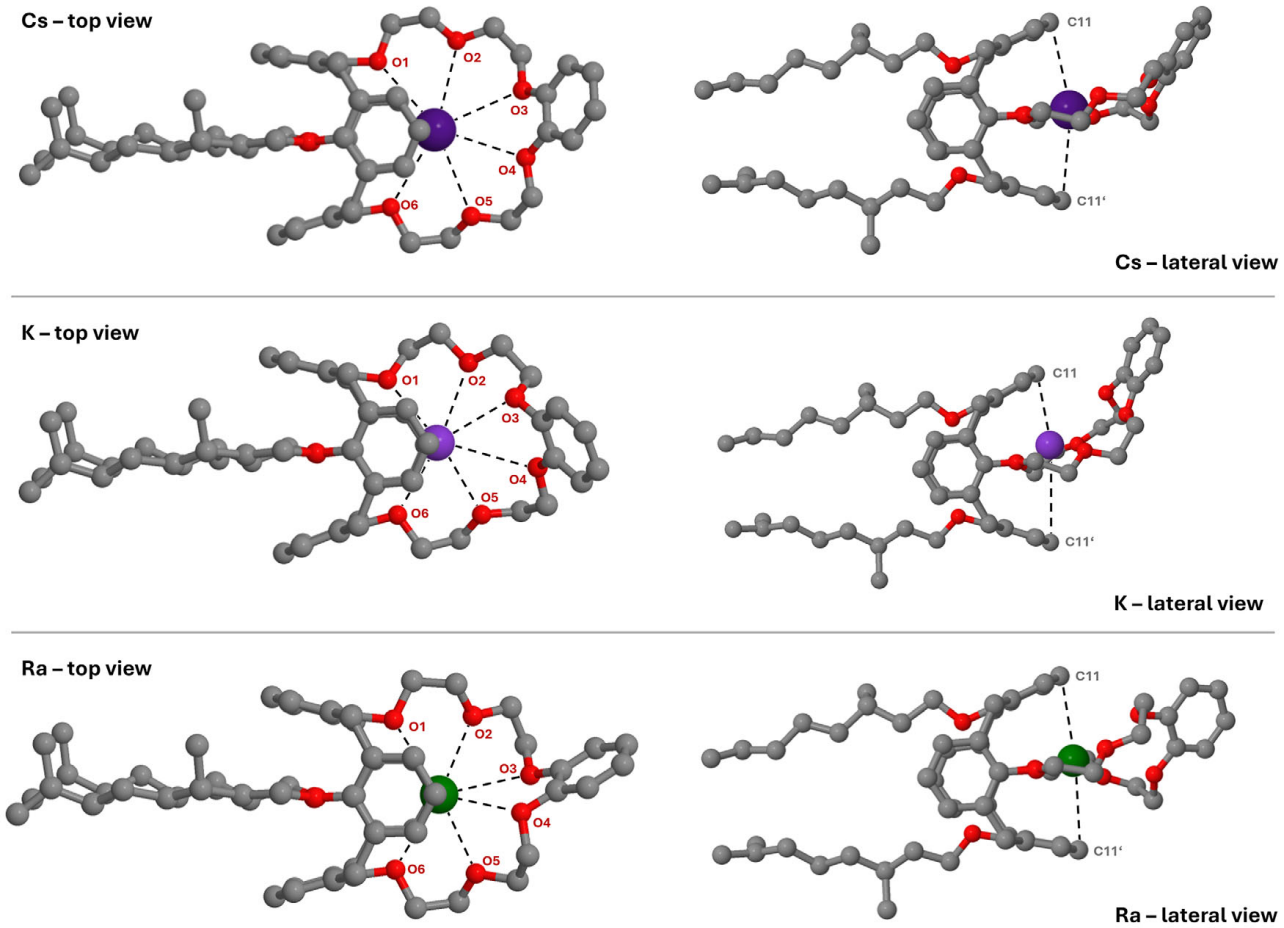
Top and lateral view of the DFT‐optimized structures of [M(MAXCalix)]^n+^ complexes (M = Cs^+^, K^+^, Ra^2+^) using BP86 functional with the def2‐TZVPP basis set for Cs^+^ and K^+^ and def‐TZVP basis set for Ra^2+^. Hydrogen atoms are omitted for visual clarity.

Based on the optimized ligand structure, the complex structures of [M(MAXCalix)]^+^ (M = Na, K, Rb, Cs, Fr) and [M(MAXCalix)]^2+^ (M = Mg, Ca, Sr, Ba, Ra) were investigated (in C1 symmetry). Figure 4 shows the complexes of Cs^+^, K^+^, and Ra^2+^ as representative examples. A comprehensive overview of all structures is provided in the (Figures S19, S20). In the Cs^+^ complex, the central cation is positioned within the 18‐crown‐ether‐6 plane and is nearly symmetrically surrounded by the oxygen atoms of the crown ether. However, as the top view of the complex indicates, the Cs^+^ ion is slightly displaced beneath the calix[4]arene rings, resulting in marginally increased bond lengths toward O3 and O4. Overall, the obtained Cs–O distances as well as the location of the Cs^+^ ion are consistent with previous studies on calix[4]arene crown ether complexes.^[^ ^22^, ^25^ ^]^ The distances between the cation and C‐11 and C‐11′ are 355.0 and 361.6 pm, respectively. This suggests the presence of π‐interactions between the individual benzene rings and the cation. The overall distance between C‐11 and C‐11′ is 708.1 pm, whereas the distance between the opposite rings, at 655.7 pm, is even further reduced compared to the unbound ligand structure. This reduction underscores the significant impact of the calix[4]arene backbone on the Cs^+^ coordination on. This also highlights the flexibility of the calix[4]arene backbone, as the inter‐arene angles are to some extent variable, as previously reported for related compounds.^[^ ^35^ ^]^ In addition, the terminal benzene ring tilts out of the coordination plane, indicating that the ligand undergoes substantial structural change to effectively bind the metal ion. The K^+^ complex shares some similarities with the Cs^+^ complex; however, the crown ether substructure undergoes a substantial change in shape. O3, O4, and the terminal benzene ring are strongly tilted out of plane formed by O1, O2, O5, and O6, leading to a half‐boat conformation. This alters the spatial orientation of the K^+^ cation, which now resides above the coordination plane. Consequently, the K–O distances range from 286.7 to 297.8 pm near the coordination site and increase significantly toward O3 and O4 (*r*_K–O3_ = 366.6 pm, *r*_K–O4_ = 368.9 pm). As the top view shows, the K^+^ ion remains slightly beneath the calix[4]arene structure, resulting in preferential interaction with one benzene ring (*r*_K–C11_ = 317.7 pm). Additionally, we performed an Energy Decomposition Analysis (EDA)^[^ ^36^ ^]^ of these alkali metal complexes, complemented by a Natural Population Analysis (NPA).^[^ ^37^ ^]^ Both methods clearly indicated that, for all four alkali metals, the interaction with the ligand is purely electrostatic, as orbital relaxation is negligible in every case. The same applies for dispersion, which remains nearly unchanged across all cases and contributes only marginally (approx. 0.6%) to the total energy. The NPA analysis further confirmed that the electron populations of the carbon and oxygen atoms ‐ those most likely to interact with the alkali metals ‐ remain nearly unchanged. Thus, these calculations support the conclusion that the metal‐ligand interactions are purely electrostatic, with no significant covalent character. We note, however, that a more thorough investigation into the bonding nature would require ab initio methods such as second‐order Møller–Plesset perturbation theory (MP2). These methods are computationally more demanding, which is why our study focused primarily on the structural aspects of the complexes.

Replacing alkali ions with alkaline earth ions significantly impacts the complex structure. The most notable change is the twisted crown ether substructure, which causes the terminal benzene ring to tilt nearly 90° relative to the coordination plane. Unlike in the K^+^ complex, the bond distances in the Ra^2+^ complex remain short, with Ra–O distances ranging from 295.4 to 298.5 pm, and Ra–O4 and Ra–O2 distances of 312.9 and 328.3 pm, respectively. The Ra^2+^ ion stays close to the coordination plane, as indicated by the nearly identical Ra‐C11/C11’ distances, but it is drawn closer to the underside of the calix[4]arene backbone compared to Cs^+^ or K^+^. Consequently, the difference in the spatial distances between the opposing benzene rings (r_C11‐C11’_ = 680.2 pm and r_C12‐C12’_ = 673.7 pm) is comparatively small. Based on these three examples, the formation of two distinct structural subtypes is evident. Subtype I features an almost planar crown ether substructure, as observed in the Cs^+^ and K^+^ complexes, while Subtype II is characterized by a twisted crown ether substructure. The complexes of Mg^2+^, Ca^2+^, Na^+^, K^+^, Rb^+^, Cs^+^, and Fr^+^ can be classified as Subtype I. Cs^+^ and Fr^+^ are similar in structure, with the cation positioned within the coordination plane. In contrast, Na^+^, K^+^, and Rb^+^ are located above the plane, causing O3 and O4 to tilt, as shown for the K^+^ complex structure in Figure 4. As the ionic radius increases, the cation is more strongly pulled into the coordination plane and further beneath the calix[4]arene rings.

Comparing the M‐O distances (given in Table 1) to the averaged M‐O in dibenzo‐18‐crown ether complexes,^[^ ^39^, ^40^, ^41^, ^42^, ^43^, ^44^, ^45^, ^46^ ^]^ an increase in coordination number from four (Na^+^, K^+^) to six (Cs^+^, Fr^+^) is observed. Rb^+^ is somewhat ambivalent, with five M‐O distances within the typical range for substantial Rb–O interaction, while the Rb–O4 distance of 355.9 pm is too elongated to be considered as a Rb─O bond. In addition, the M‐C11 and M‐C11’ distances suggest π‐interactions between the calix[4]arene rings facing the metal ion. For the smaller ions (Na^+^, K^+^, Rb^+^), only one significant interaction occurs, while for the larger ions (Cs^+^, Fr^+^), two interactions occur due to the more symmetrical coordination environment.

**Table 1:** Summary of M–O and M‐C11/C11’ distances in the [M(MAXCalix)]^+^ (M = Na, K, Rb, Cs, Fr) and [M(MAXCalix)]^2+^ (M = Mg, Ca, Sr, Ba, Ra) based on the DFT‐optimized structures. Structures are displayed in Figure 4, Figures S19, S20 in the Supporting Information. The complexes can be assigned to distinct subtypes with Subtype I representing an almost planar crown ether and Subtype II representing a twisted crown ether substructure. Distances given in pm.

	*r*_M_^[^ ^38^ ^]^	M–O1	M–O2	M–O3	M–O4	M–O5	M–O6	M–C11	M–C11’	Subtype
Na^+^	102	313.9	253.6	283.2	266.1	259.3	376.6	275.5	550.2	I
K^+^	138	286.7	299.2	366.6	368.9	297.8	292.8	317.7	426.8	I
Rb^+^	152	310.0	316.5	331.1	355.9	319.0	311.9	334.3	397.2	I
Cs^+^	167	320.3	332.8	344.8	349.0	336.5	318.8	355.0	361.6	I
Fr^+^	180	323.3	335.8	344.9	347.4	337.6	322.0	362.6	364.3	I
Mg^2+^	72	324.9	215.9	226.5	319.6	211.1	442.4	265.8	627.5	I^[a]^
Ca^2+^	100	280.3	249.1	270.1	256.8	243.5	363.7	277.3	600.6	I^[a]^
Sr^2+^	118	321.9	260.1	290.6	279.4	262.7	271.7	295.7	479.4	II
Ba^2+^	135	294.4	283.7	331.1	307.8	281.5	288.3	321.6	360.2	II
Ra^2+^	148	295.4	298.5	328.3	312.9	293.4	295.0	332.9	348.2	II

^[a]^ Strongly distorted; complex only obtained with preorganized structure based on the Na^+^ complex, otherwise no complex formation observed.

Mg^2+^ and Ca^2+^ also form Subtype I complexes. However, NMR, ESI^+^ MS, and extraction studies have demonstrated that these ions do not form stable complexes with MAXCalix. In the gas phase, the corresponding structures were obtained only by substituting Na^+^ in a pre‐optimized Na^+^ structure with Mg^2+^ or Ca^2+^. The resulting complexes show significant distortion in both the calix[4]arene backbone and the crown ether, reflecting the unfavorable nature of these interactions. Thus, complexation is only possible under artificial conditions, as calculations reveal no interaction between Mg^2+^ or Ca^2+^ and the ligand when the ion is merely close to the ligand. This behavior is in contrast with all other ions tested, which readily transition into the coordination pocket of MAXCalix.

Contrary to Mg^2+^ and Ca^2+^, the larger alkaline earth ions Sr^2+^, Ba^2+^, and Ra^2+^ form Subtype II complexes. As the ionic radius increases, the central ion is more strongly drawn into the coordination plane, resulting in a more symmetrical arrangement of the calix[4]arene rings. The twisted crown ether structure ensures four‐fold oxygen coordination, with average M–O distances of 287 pm for Ba^2+^ and 295 pm for Ra^2+^, which align well with the M–O distances reported for crown ether complexes in the literature.^[^ ^47^, ^48^ ^]^

Taking previous extraction data and the here presented data into account, it is evident that the increased affinity of MAXCalix toward larger ions originates from a more symmetrical coordination environment of the cation in addition to a stabilizing effect through π‐interactions with the calix[4]arene backbone. This interaction is evident from NMR shifts in the proton and carbon data, since the difference in the benzene ring shifts are correlated to the size of the ion and therefore, to the complexation strength.

## 3 Conclusion

In this work, we demonstrated that the coordination of the calix[4]arene crown ether, MAXCalix, is highly dependent on the ionic radius of the metal ion. While MAXCalix effectively coordinates large ions like Cs^+^, it can also form complexes with smaller ions such as Na^+^, underscoring the versatility of this ligand and calix[4]arene crown ethers in general. The ion size dictates the complex structure, with two distinct structural subtypes identified through NMR and DFT calculations. In addition, π‐interactions between the cation and the metal‐facing benzene rings of the calix[4]arene backbone significantly enhance the stability of the complexes. Larger ions, such as Cs^+^, benefit from π‐interactions with both metal‐facing rings, while smaller ions like K^+^ benefit only from one such interaction, if at all. Consequently, these interactions are primarily responsible for the enhanced affinity toward Cs^+^, making MAXCalix an effective and selective extractant.

The data presented here complements existing structural and theoretical studies on Cs^+^ complexation with calix[4] crown ethers. Moreover, by systematically exploring the coordination chemistry of elements from the first and second main groups of the periodic table, this work significantly enhances our understanding of the underlying interaction mechanisms. This knowledge can be used to improve existing extraction protocols or to develop new procedures for radionuclide decontamination and related applications.

## Supporting Information

Electronic Supporting Information (ESI) available: Synthetic and theoretical procedures, NMR spectra, ESI^+^ MS spectra, DFT structures. The authors have cited additional references within the Supporting Information.^[^ ^49^, ^50^, ^51^, ^52^, ^53^, ^54^, ^55^, ^56^, ^57^, ^58^, ^59^, ^60^, ^61^, ^62^, ^63^ ^]^

## Conflict of Interests

The authors declare no conflict of interest.

## Supporting information



Supporting Information

## Data Availability

The data that support the findings of this study are available in the supporting information of this article.
