# Supplementary material for: The Role of Ion Size and π‐Interaction in Stabilizing Calix[4]arene Crown Ether Metal Complexes
Source: Chemistry. 2025 May 21;31(40):e202501065. doi: 10.1002/chem.202501065 (PMC12271986; doi:10.1002/chem.202501065)
Supplement: Supplementary file 1 — Supporting Information [file CHEM-31-e202501065-s001.docx]

**Supplementary Information for**

The Role of Ion Size and π-Interaction in stabilizing Calix[4]arene Crown Ether Metal Complexes

Thomas Sittel,*^[a]^ Karolin Becker, ^[b]^ Robert Polly, ^[a]^ Udo Müllich, ^[a]^ Andreas Geist, ^[a]^ and Petra J. Panak^[a,b]^

[a] Dr. T. Sittel, Dr. R. Polly, U. Müllich, Dr. A. Geist, Prof. Dr. P. J. Panak
Karlsruhe Institute of Technology
Institute for Nuclear Waste Disposal
P.O. Box 3640, 76021 Karlsruhe, Germany
E-mail: thomas.sittel@kit.edu

[b] K. Becker, Prof. Dr. P. J. Panak
Heidelberg University
Institute for Physical Chemistry
Im Neuenheimer Feld 253, 69120 Heidelberg, Germany

**Table of Contents**

[Methods and materials 2](#_Toc190857153)

[NMR sample preparation 2](#_Toc190857154)

[Competitive NMR studies 3](#_Toc190857155)

[Single-metal ion speciation studies 4](#_Toc190857156)

[NMR data 5](#_Toc190857157)

[MAXCalix 5](#_Toc190857158)

[[Cs(MAXCalix)]OTf 7](#_Toc190857159)

[[Rb(MAXCalix)]OTf 9](#_Toc190857160)

[[K(MAXCalix)]OTf 10](#_Toc190857161)

[[NH_4_(MAXCalix)]OTf 12](#_Toc190857162)

[[Na(MAXCalix)]OTf 13](#_Toc190857163)

[[Sr(MAXCalix)]OTf_2_ 15](#_Toc190857164)

[Competitive Speciation Study 17](#_Toc190857165)

[DFT structures 19](#_Toc190857166)

[References 21](#_Toc190857167)

## Methods and materials

MAXCalix has been obtained from Marshallton Research Laboratories, King, North Carolina, USA. Metal salts and deuterated solvents were purchased from Sigma Aldrich, Merck, Alfa Aesar and Euriso-top GmbH. Cs(CF_3_SO_3_), Ca(CF_3_SO_3_)_2_, Rb(CF_3_SO_3_), and Sr(CF_3_SO_3_)_2_ solutions were prepared by dissolving CsOH, CaCO_3_, Rb_2_CO_3_,and SrCO_3_ in 0.07 M trifluoromethanesulfonic acid (HOTf).

NMR spectra were recorded on a Bruker Avance III 400 spectrometer operating at 400.13 MHz for ^1^H, 100.63 MHz for ^13^C and 40.58 MHz for ^15^N at 300 K. The spectrometer was equipped with a broadband observe probe (BBFOplus) with direct x-magnetization detection for proton and heteronuclear detection experiments. Chemical shifts are referenced internally to TMS (δ(TMS) = 0 ppm) for ^1^H and ^13^C and to CFCl_3_ with δ(C^19^FCl_3_) = 0 ppm for ^19^F. For all spectra, standard Bruker pulse sequences were used. 1D spectra of ^1^H and ^13^C were recorded with 32k data points and are zero filled to 64k data points. ^19^F NMR data were recorded with 64k data points. Signal multiplicity was determined as s (singlet), d (doublet), t (triplet), q (quartet), quin (quintet), sex (sextet), sept (septet), m (multiplet) and br. s (broad signal).

Mass spectra using ESI ionization methods were recorded on a Bruker ApexQe FT ICR instrument.

Quantum chemical calculations on the MAXCalix extracting agent were performed and the structures of the complexes with the incorporated alkali (Na^+^, K^+^, Rb^+^, Ca^+^, Fr^+^) and earth alkali metals (Mg^2+^, Ca^2+^, Sr^2+^, Ba^2+^, Ra^2+^) were determined theoretically. We employed DFT^1, 2^ calculations using the BP86 functional,^3^ with the def2-TZVPP^4, 5^ basis set (with the exception of and Fr^+^ and Ra^2+^ where we used the def-TZVP^6^ basis set). We optimized the structures of the isolated MAXCalix extracting agent and all the complexes with alkali and earth-alkaline cations with by DFT approach. Additionally, we determined the vibrational frequencies of the optimized structures. Calculations were performed using TURBOMOLE (version 7.0, 2015).^4-12^ This theoretical set up proved to have high accuracy in several earlier studies.^13-17^

### NMR sample preparation

Variation of ligand concentration

10 µmol M(OTf)_x_ (M = Na, K, Rb, Cs, Mg, Ca, Sr, NH_4_; x = 1-2) were dissolved in 480 µL acetone-d_6_ and transferred into an NMR tube. In addition, 20 µmol MAXCalix were dissolved in 240 µL deuterated solvent. Then the MAXCalix solution was added stepwise (2.5 µmol = 30 µL per step) to the metal salt solution. After shaking the NMR tube, the complexation progress was followed by collecting ^1^H and ^19^F NMR data.

Variation of metal salt concentration

10 µmol MAXCalix were dissolved in 400 µL deuterated acetone-d_6_ and transferred into an NMR tube. 20 µmol M(OTf)_x_ (M = Na, K, Rb, Cs, Mg, Ca, Sr, NH_4_; x = 1-2) were dissolved in 400 µL acetone-d_6_, and this solution was added stepwise to the ligand solution (2 µmol = 40 µL per step). The NMR tube was then rigorously shaken, and the complexation progress was followed by collecting ^1^H and ^19^F data.

### Competitive NMR studies

Competitive NMR studies between ions pairs were carried out in a 3-way-progress.

1. 10 µmol of each metal salt were dissolved in 240 µL deuterated solvent each and then transferred into an NMR tube. 30 µmol MAXCalix were dissolved in 360 µL deuterated solvent and the solution was added stepwise (2.5 µmol = 20 µL) to the metal salt mixture. The complexation was then followed by collecting ^1^H and ^19^F NMR data.
2. 10 µmol MAXCalix was dissolved in 480 µL acetone-d_6_ and added to metal salt M1 (10 µmol), forming the complex [M1(MAXCalix)]^+^. Then 20 µmol of the metal salt M2 was dissolved in 400 µL acetone-d_6_ and the solution was added stepwise to the complex solution (2 µmol = 40 µL). The progress was followed by collecting ^1^H and ^19^F NMR data.
3. 10 µmol MAXCalix was dissolved in 480 µL acetone-d_6_ and added to metal salt M2 (10 µmol), forming the complex [M2(MAXCalix)]^+^. Then 20 µmol of the metal salt M1 was dissolved in 400 µL acetone-d_6_ and added stepwise to the complex solution (2 µmol = 40 µL). The progress was followed by collecting ^1^H and ^19^F NMR data.

## Single-metal ion speciation studies


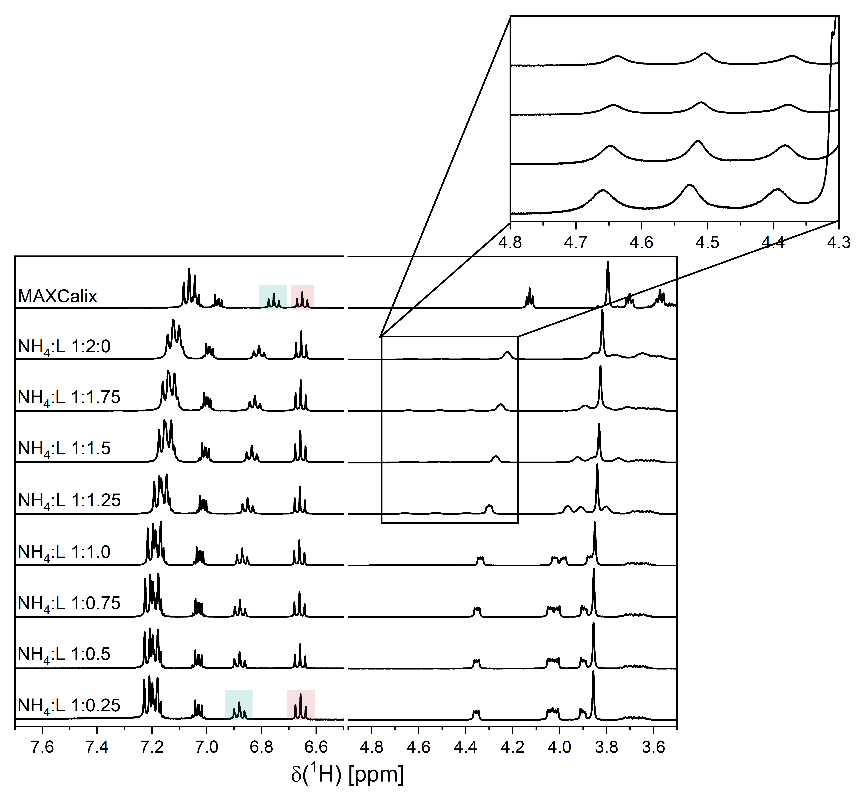

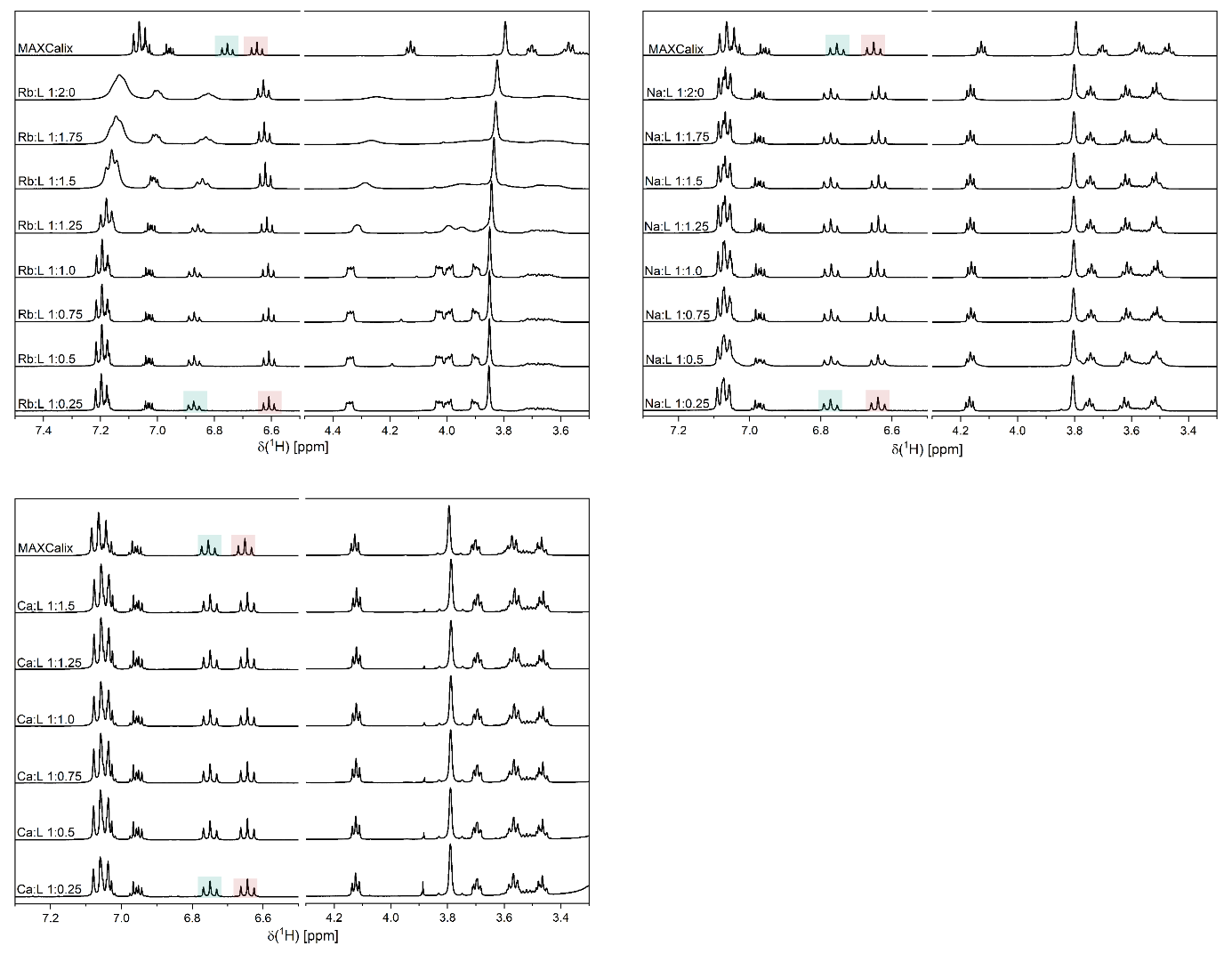


**Figure S1.** ^1^H-NMR spectra (400.13 MHz, 300 K) of the aromatic and crown ether region of MAXCalix depending on the MAXCalix/metal ion ratio for Rb^+^, Na^+^, Ca^2+^, and NH_4_^+^ ([M] = 1.7∙10^-2^ mol L^-1^) in acetone-d_6_. ■ H-11, ■ H-12.

## NMR data

###
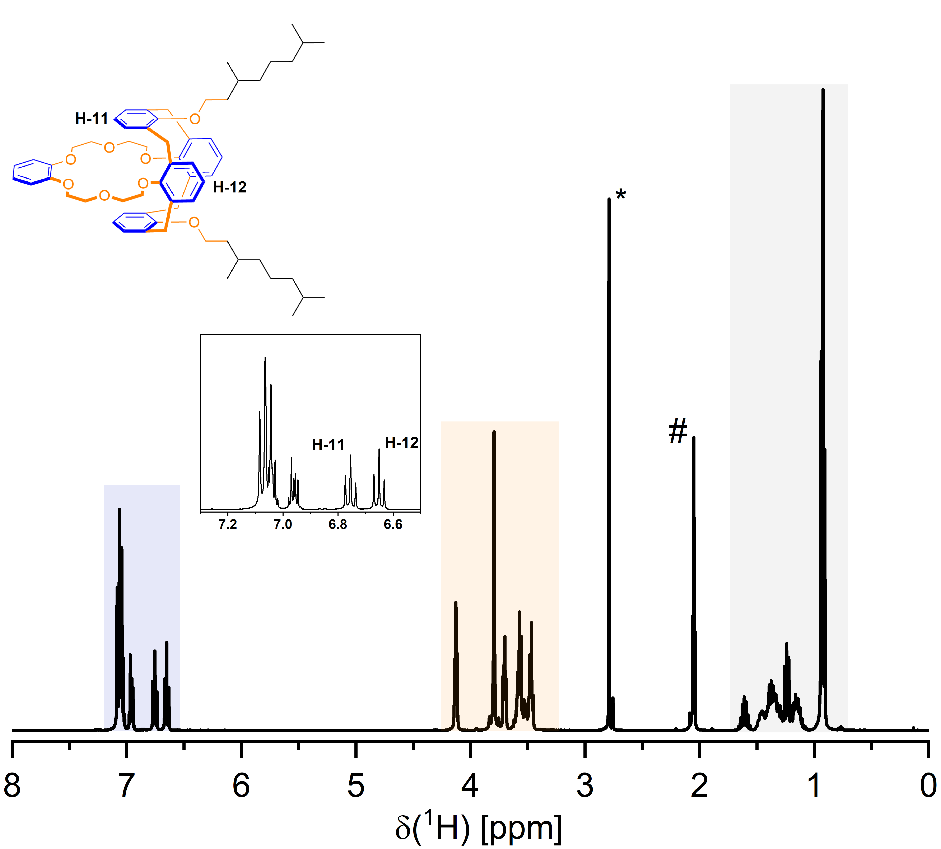

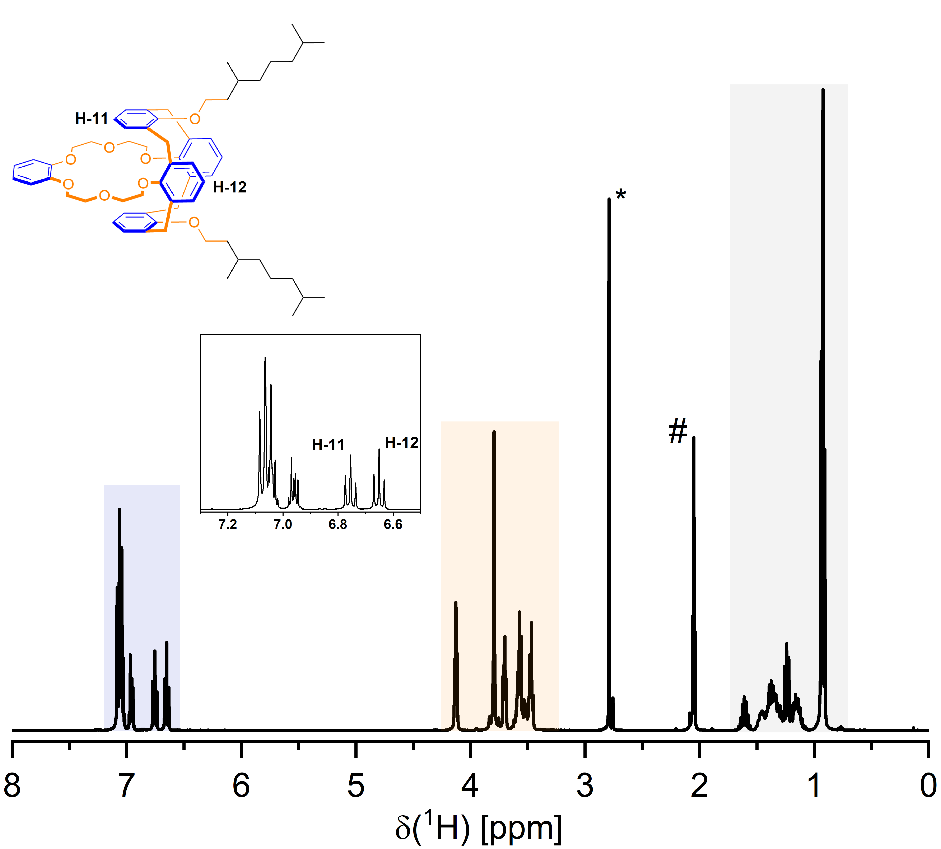
MAXCalix

**Figure S2.** ^1^H-NMR spectrum (400.17 MHz, 300K) of MAXCalix in Acetone-d_6_ highlighting the different structure subtypes of the calix[4]arene crown ether. * δ(H_2_O). # δ(Acetone).

This section discusses the proton NMR spectrum of MAXCalix in acetone-d_6_ as a guideline for the subsequent complexation studies. The ligand can be divided into three substructures: the aromatic region, which includes the calix[4]arene backbone and the benzene ring attached to the crown ether; the 18-crown-6-ether; and the alkyl moieties, which primarily contribute to solubility. As highlighted in the figure, the proton NMR spectrum clearly reflect these distinct substructures. The aromatic protons appear in the δ = 6.5 – 7.5 ppm range (blue). A notable and important feature in the aromatic region is the well-separated triplets at 6.6 ppm and 6.7 pm. These triplets correspond to the terminal protons H11 and H12 of the calix[4]arene backbone. The crown ether region spans a chemical shift range of δ = 3.5 – 4.5 ppm (orange). This region includes four triplets representing the CH_2_ groups of the crown ether. Additionally, the singlet corresponds to the bridging CH_2_ groups of the calix[4]arene backbone. The second aliphatic region (δ = 1.0 – 1.5 ppm, grey) is assigned to the protons of the 3,7-dimethyloctyl chain. These alkyl moieties are primarily important for enhancing solubility in non-polar solvents and do not participate in the complexation.


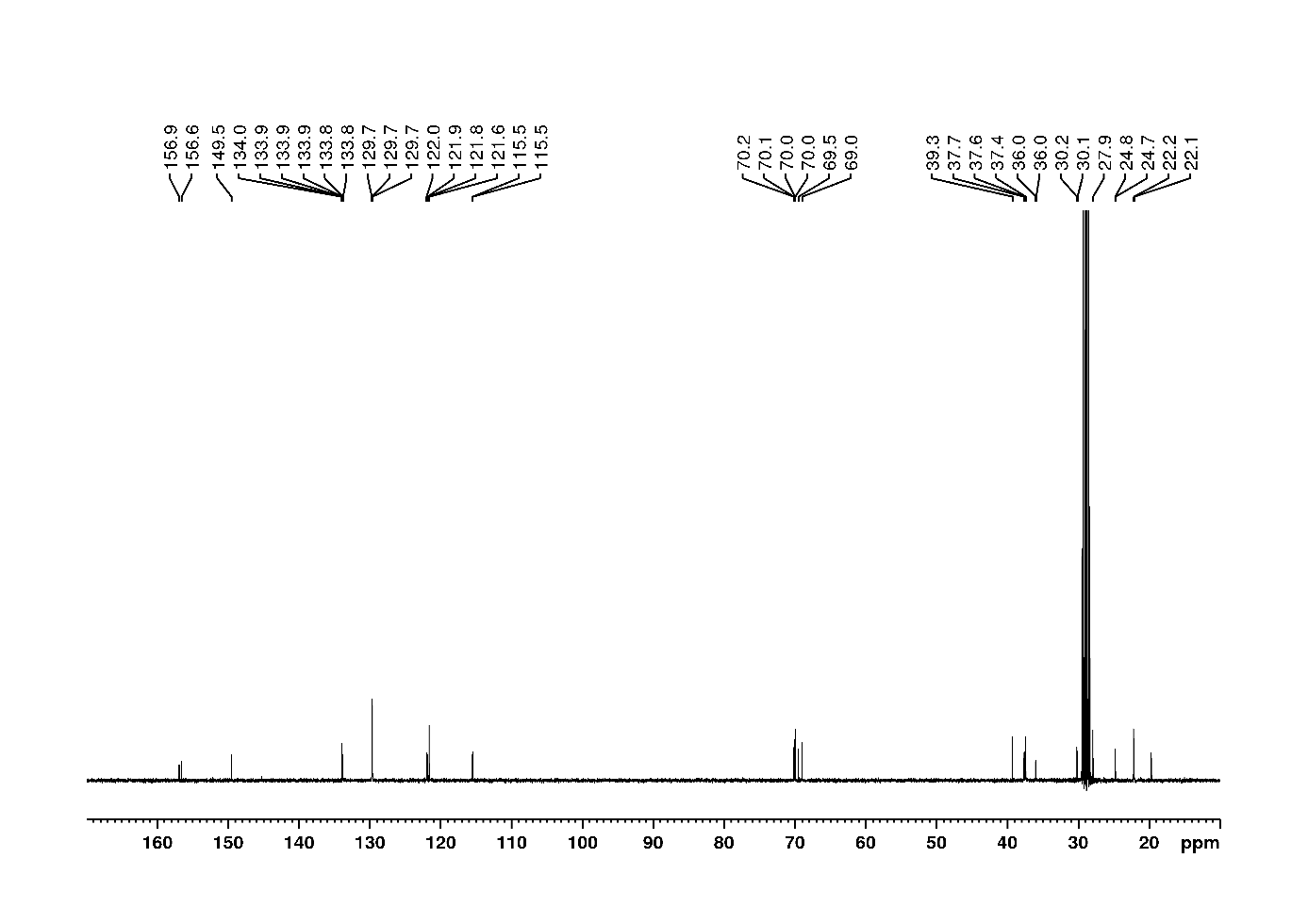


**Figure S3.** ^13^C spectrum (100.63 MHz, 300K) of MAXCalix in acetone-d_6_.

**^1^H NMR** (400 MHz, d-Aceton, 300K): δ[ppm]= 7.11 – 7.01 (m, 10H, H-2,10,13), 6.98 – 6.92 (m, 2H, H-1), 6.75 (t, *J* = 7.5 Hz, 2H, H-11), 6.65 (t, *J* = 7.5 Hz, 2H, H-12), 4.12 (t, *J* = 5.0 Hz, 4H, H-4-7,17), 3.79 (s, 8H, H-15), 3.70 (td, *J* = 5.0, 1.6 Hz, 4H, H-4-7,17), 3.64 – 3.43 (m, 12H, H-4-7,17), 1.68 – 1.53 (m, 2H, H-23), 1.51 – 1.08 (m, 18H, H18-22), 0.99 – 0.86 (m, 18H, H-24,25).

**^13^C NMR** (101 MHz, d-Aceton, 300K): δ[ppm] = 156.9 (C_q_, C-8/16), 156.6 (C_q_, C-8/16), 149.5 (C_q_, C-3), 133.9 (C_q_, C-9/14), 133.8 (C_q_, C-9/14), 129.7 (CH, C-10/13), 129.7 (CH, C-10/13), 122.0 (CH, C-1/11/12), 121.9 (CH, C-1/11/12), 121.6 (CH, C-1/11/12), 115.5 (CH, C-2), 70.2 (CH_2_, C-4/5/6/7/17), 70.0 (CH_2_, C-4/5/6/7/17), 70.0 (CH_2_, C-4/5/6/7/17), 69.5 (CH_2_, C-4/5/6/7/17), 69.0 (CH_2_, C-4/5/6/7/17), 39.3 (CH_2_, C-18/19/20/21/22), 37.7 (CH_2_, C-18/19/20/21/22) 37.4 (CH_2_, C-15), 36.0 (CH_2_, C-18/19/20/21/22), 30.2 (CH_2_, C-18/19/20/21/22), 27.9 (CH_2_, C-23),, 24.8 (CH_2_, C-18/19/20/21/22), 22.2 (CH_3_, C-25), 19.7 (CH_3_, C-24).

### [Cs(MAXCalix)]OTf


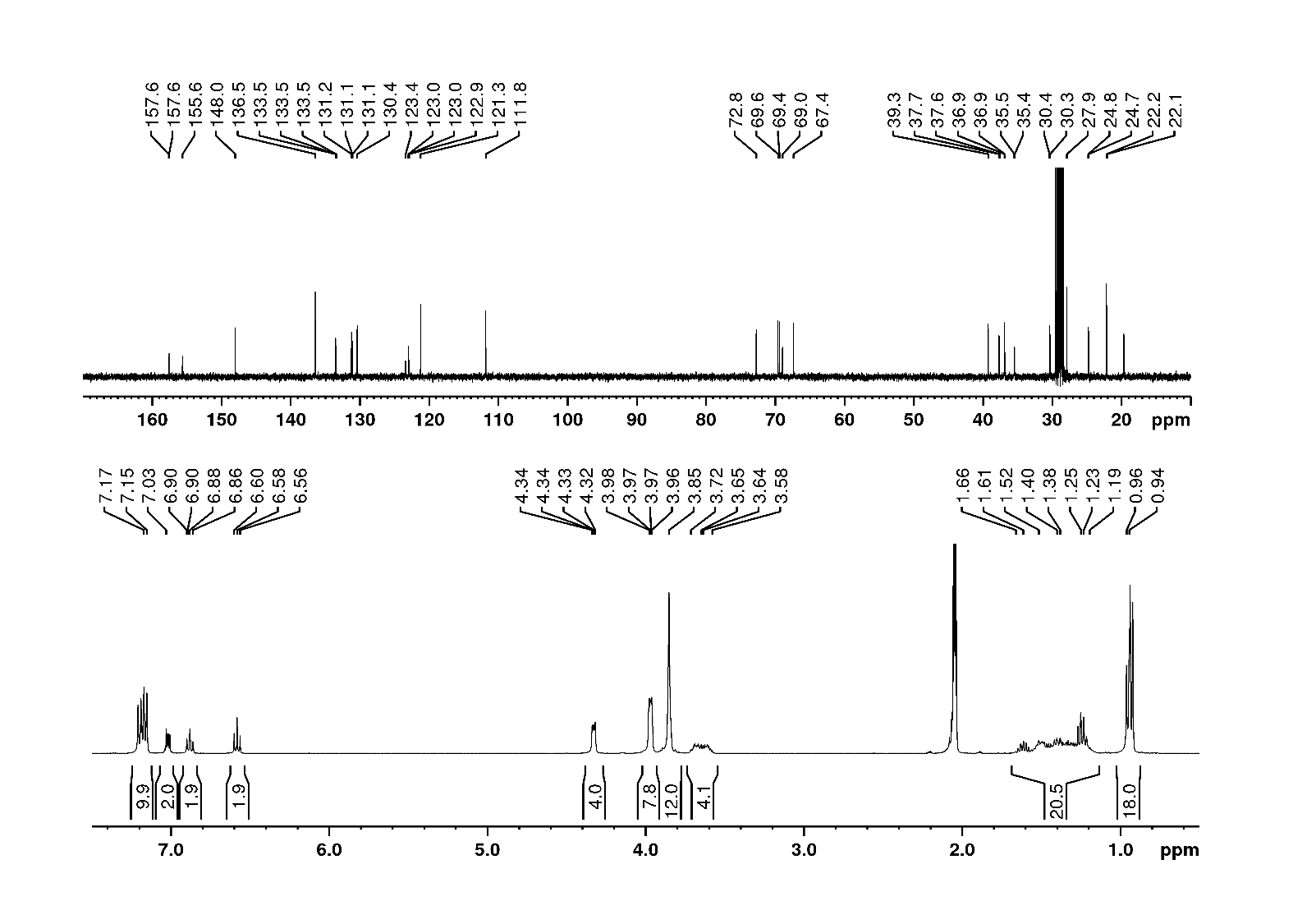


**Figure S4.** ^1^H (bottom, 400.17 MHz, 300K) and ^13^C (top, 100.63 MHz, 300K) spectrum of [Cs(MAXCalix)]OTf in acetone-d_6_.


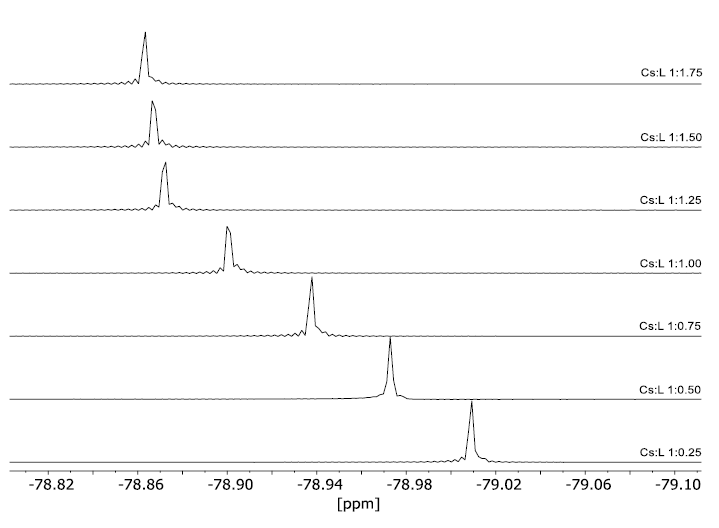


**Figure S5.** Evolution of the ^19^F-NMR spectra (376.50 MHz, 300 K) of CF_3_SO_3_^-^ anion depending on the MAXCalix/Cs^+^ ratio in acetone-d_6_.


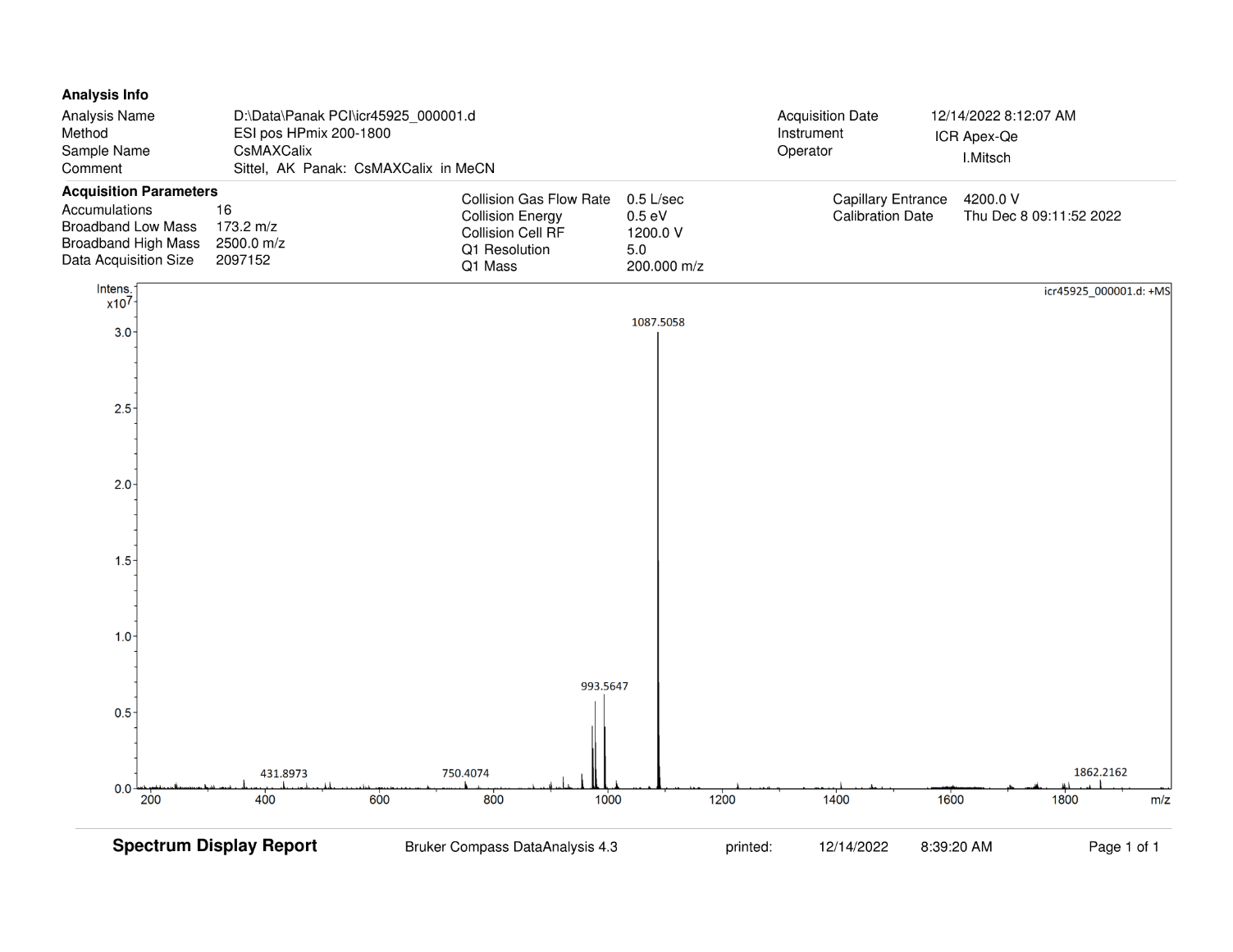


**Figure S6.** ESI^+^ mass spectrum of [Cs(MAXCalix)]^+^.

**^1^H NMR** (400.17 MHz, d-Aceton, 300K): δ[ppm] = 7.23 – 7.13 (m, 10H, H-2,10,13), 7.05 – 6.98 (m, 2H, H-1), 6.88 (t, *J*=7.5, 2H, H-11), 6.58 (t, *J*=7.5, 2H, H-12), 4.36 – 4.30 (m, 4H, H-4), 4.01 – 3.94 (m, 8H, H-15), 3.86 (s, 12H, H-5-7/17), 3.74 – 3.56 (m, 4H, H-5-7/17), 1.68 – 1.14 (m, 20H, H-18-23), 1.01 – 0.88 (m, 18H, H-24,25).

**^13^C NMR** (100.63 MHz, d-Aceton, 300K): δ[ppm] = 157.6 (C_q_, C-8/16), 155.6 (C_q_, C-8/16), 148.0 (C_q_, C-3), 136.5 (C_q_, C-9/14), 133.5 (C_q_, C-9/14), 131.2 (CH, C-10/13), 130.4 (CH, C-10/13), 123.4 (CH, C-1/11/12), 123.0 (CH, C-1/11/12), 121.3 (CH, C-1/11/12), 111.8 (CH, C-2), 72.8 (CH_2_, C-5/6/7/17), 69.6 (CH_2_, C-5/6/7/17), 69.4 (CH_2_, C-5/6/7/17), 69.0 (CH_2_, C-5/6/7/17), 67.4 (CH_2_, C-4), 39.3 (CH_2_, C-18/19/20/21/22), 37.7 (CH2, C-15), 35.5 (CH_2_, C-18/19/20/21/22), 30.4 (CH_2_, C-18/19/20/21/22), 27.9 (CH_2_, C-18/19/20/21/22), 27.9 (CH_2_, C-23), 24.8 (CH_2_, C-18/19/20/21/22), 22.2 (CH3, C-25), 19.7 (CH3, C-24).

**^19^F NMR** (376.50 MHz, d-Aceton, 300K): δ[ppm] = -78.94 (CF_3_SO_3_^-^).

**MS (ESI^+^):** [M^+^] = CsC_62_H_82_O_8_, calculated: 1087.5064, found: 1087.5058.

### [Rb(MAXCalix)]OTf


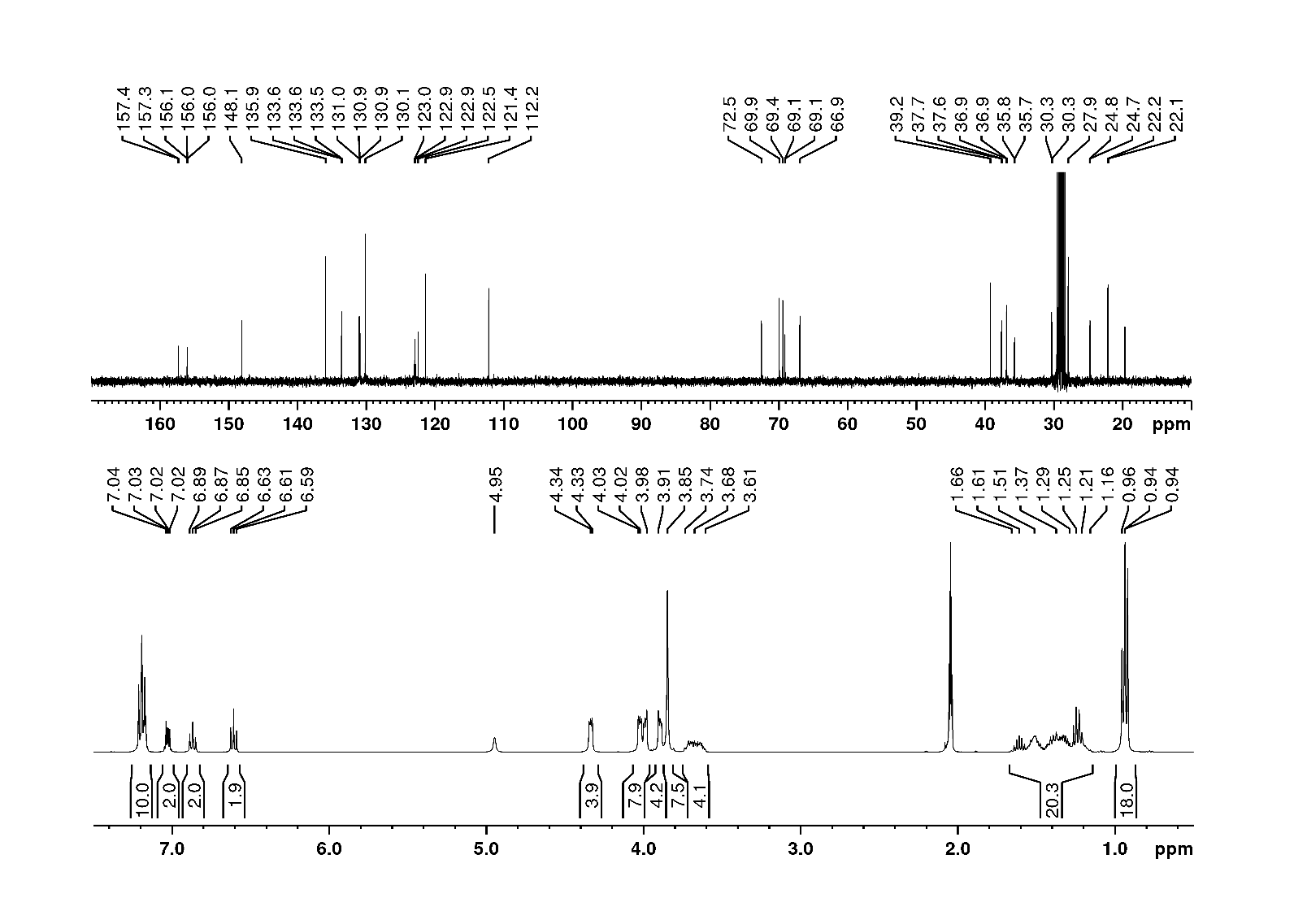


**Figure S7.** ^1^H (bottom, 400.17 MHz, 300K) and ^13^C (top, 100.63 MHz, 300K) spectrum of [Rb(MAXCalix)]OTf in acetone-d_6_.


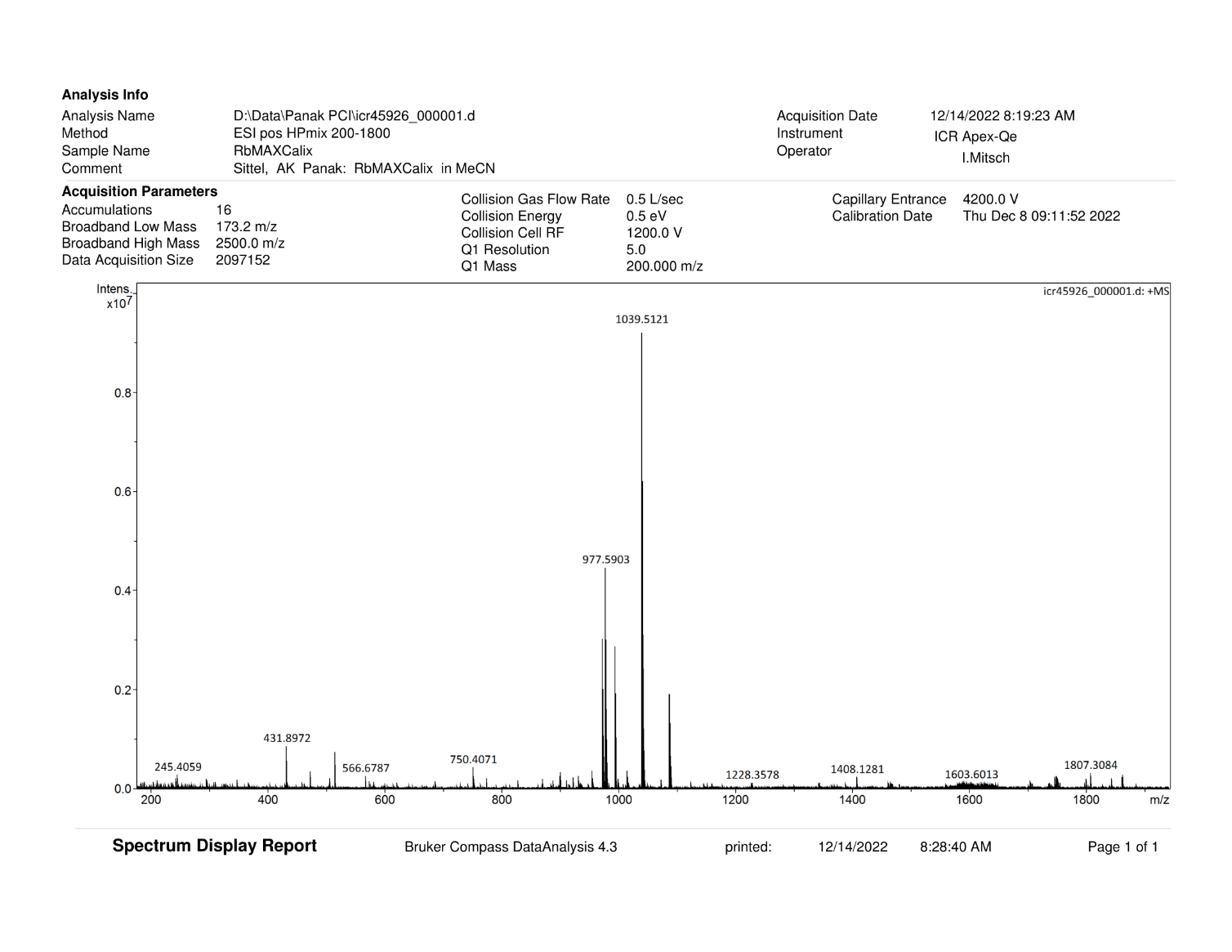


**Figure S8.** ESI^+^ mass spectrum of [Rb(MAXCalix)]^+^.

**^1^H NMR** (400.17 MHz, d-Aceton, 300K): δ[ppm] = 7.25 – 7.14 (m, 10H, H-2,10,13), 7.06 – 6.99 (m, 2H, H-1), 6.87 (td, J=7.5, 1.6, 2H, H-11), 6.61 (t, J=7.5, 2H, H-12), 4.37 – 4.30 (m, 4H, H-4), 4.06 – 3.96 (m, 8H, H-5-7/17), 3.93 – 3.87 (m, 4H, H-5-7/17), 3.85 (s, 8H, H-15), 3.76 – 3.58 (m, 4H, H-5-7/17), 1.68 – 1.14 (m, 20H, H-18-23), 0.98 – 0.90 (m, 18H, H-24,25).

**^13^C NMR** (100.63 MHz, d-Aceton, 300K): δ[ppm] = 157.3 (C_q_, C-8/16), 156.0 (C_q_, C-8/16), 148.1 (C_q_, C-3), 135.9 (C_q_, C-9/14), 133.6 (C_q_, C-9/14), 131.0 (CH, C-10/13), 130.2 (CH, C-10/13), 122.9 (CH, C-1/11/12), 122.5 (CH, C-1/11/12), 121.4 (CH, C-1/11/12), 112.2 (CH, C-2), 72.5 (CH_2_, C-5/6/7), 69.9 (CH_2_, C-5/6/7), 69.4 (CH_2_, C-17), 69.1 (CH_2_, C-5/6/7), 66.9 (CH_2_, C-4), 39.2 (CH_2_, C-18/19/20/21/22), 37.7 (CH_2_, C-18/19/20/21/22), 36.9 (CH2, C-15), 35.7 (CH_2_, C-18/19/20/21/22), 30.3 (CH_2_, C-18/19/20/21/22), 27.9 (CH_2_, C-23), 24.8 (CH_2_, C-18/19/20/21/22), 22.2 (CH3, C-25), 19.7 (CH3, C-24).

**^19^F NMR** (376.50 MHz, d-Aceton, 300K): δ[ppm] = -78.78 (CF_3_SO_3_^-^).

**MS (ESI^+^):** [M^+^] = RbC_62_H_82_O_8_, calculated: 1039.5128, found: 1039.5121.

### [K(MAXCalix)]OTf


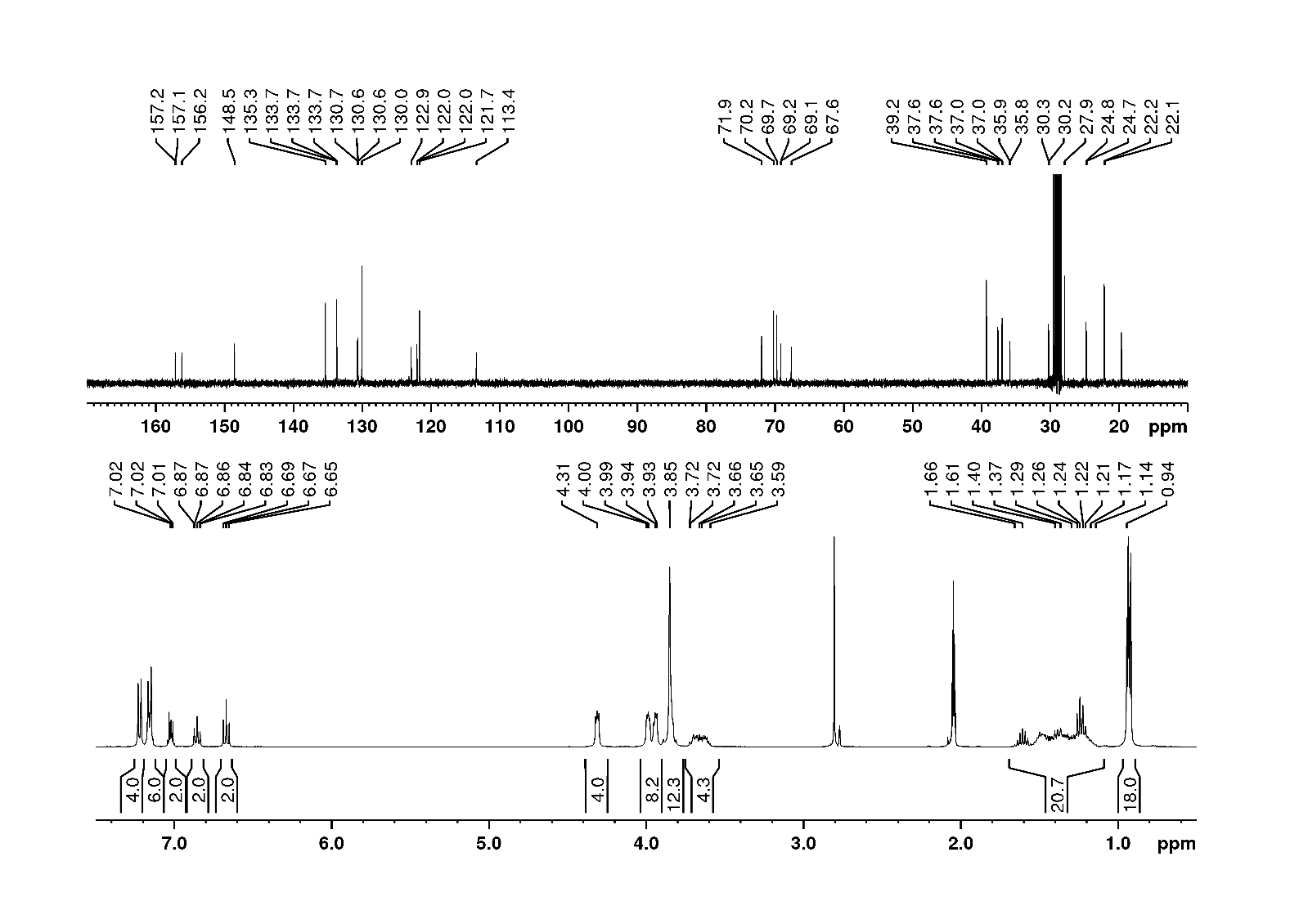


**Figure S9.** ^1^H (bottom, 400.17 MHz, 300K) and ^13^C (top, 100.63 MHz, 300K) spectrum of [K(MAXCalix)]OTf in acetone-d_6_.


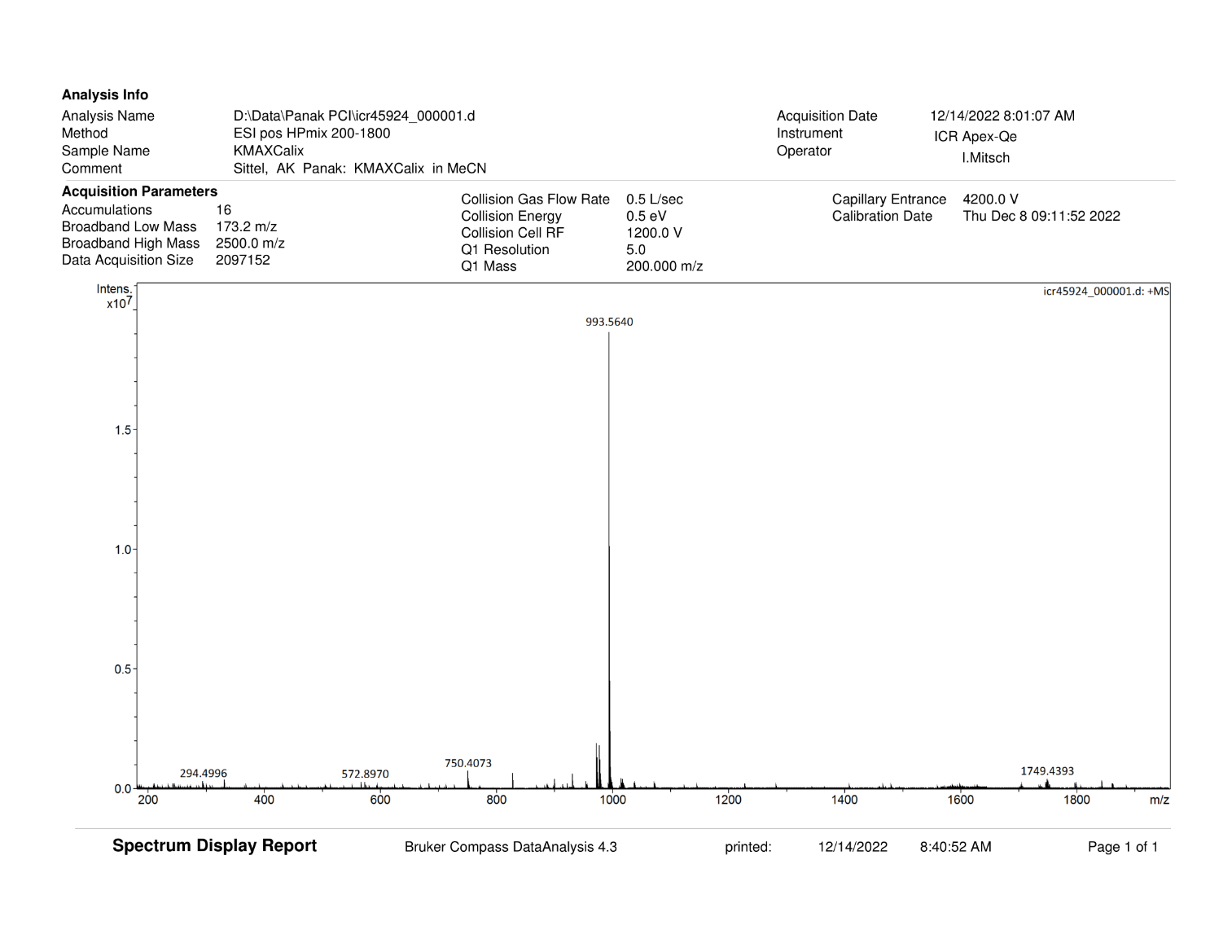


**Figure S10.** ESI^+^ mass spectrum of [K(MAXCalix)]^+^.

**^1^H NMR** (400.17 MHz, d-Aceton): δ[ppm] = 7.22 (d, *J*=7.5, 4H, H-13/10), 7.19 – 7.11 (m, 6H, H-2,10/13), 7.07 – 6.98 (m, 2H, H-1), 6.85 (t, *J*=7.5, 2H, H-11), 6.67 (t, *J*=7.5, 2H, H-12), 4.35 – 4.28 (m, 4H, H-4-7,17), 4.02 – 3.91 (m, 8H, H-15), 3.90 – 3.80 (m, 12H, H-4-7/17), 3.74 – 3.57 (m, 4H, H-4-7/17), 1.66 – 1.13 (m, 20H, H-18-23), 0.98 – 0.88 (m, 18H, H-24,25).

**^13^C NMR** (100.63 MHz, d-Aceton, 300K): δ[ppm] = 157.1 (C_q_, C-8/16), 156.2 (C_q_, C-8/16), 148.5 (C_q_, C-3), 135.3 (C_q_, C-9/14), 133.7 (C_q_, C-9/14), 130.7 (CH, C-10/13), 130.0 (CH, C-10/13), 122.9 (CH, C-1/11/12), 122.0 (CH, C-1/11/12), 121.7 (CH, C-1/11/12), 113.4 (CH, C-2), 71.9 (CH_2_, C-5/6/7/17), 70.2 (CH_2_, C-5/6/7/17), 69.7 (CH_2_, C-5/6/7/17), 69.2 (CH_2_, C-5/6/7/17), 67.6 (CH_2_, C-4), 39.2 (CH_2_, C-18/19/20/21/22), 37.6 (CH_2_, C-18/19/20/21/22), 37.0 (CH2, C-15), 35.8 (CH_2_, C-18/19/20/21/22), 30.3 (CH_2_, C-18/19/20/21/22), 27.9 (CH_2_, C-23), 24.8 (CH_2_, C-18/19/20/21/22), 22.2 (CH3, C-25), 19.7 (CH3, C-24).

**^19^F NMR** (376.50 MHz, d-Aceton, 300K): δ[ppm] = -78.78 (CF_3_SO_3_^-^).

**MS (ESI^+^):** [M^+^] = KC_62_H_82_O_8_, calculated: 993.5647, found: 993.5640.

### [NH_4_(MAXCalix)]OTf


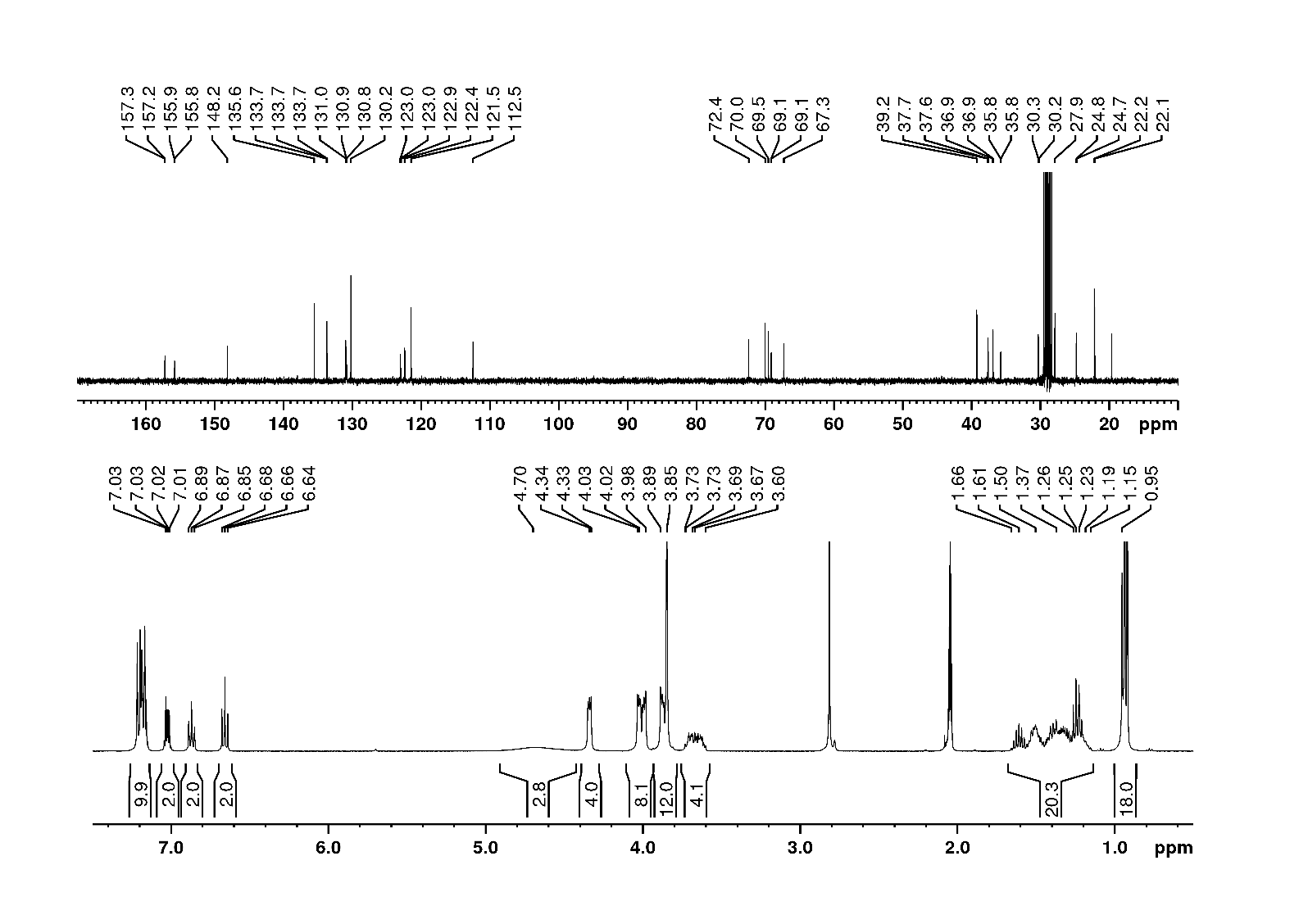


**Figure S11.** ^1^H (bottom, 400.17 MHz, 300K) and ^13^C (top, 100.63 MHz, 300K) spectrum of [NH_4_(MAXCalix)]OTf in acetone-d_6_.


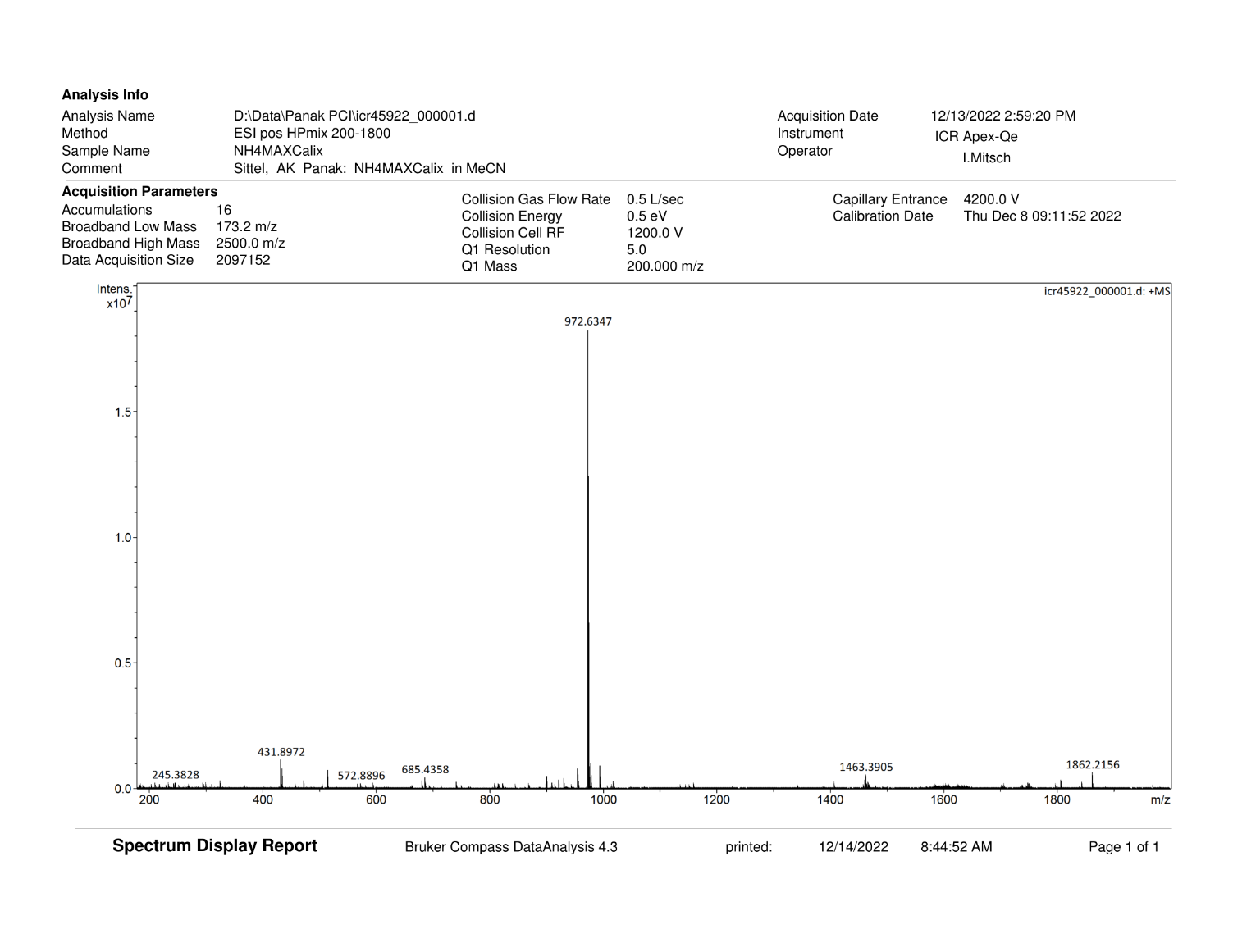


**Figure S12.** ESI^+^ mass spectrum of [NH_4_(MAXCalix)]^+^.

**^1^H NMR** (400.17 MHz, d-Aceton, 300K): δ[ppm] = 7.25 – 7.13 (m, 10H, H-2,10,13), 7.05 – 6.99 (m, 2H, H-1), 6.87 (td, *J*=7.5, 1.7, 2H, H-11), 6.66 (t, *J*=7.5, 2H, H-12), 4.37 – 4.31 (m, 4H, H-4), 4.06 – 3.96 (m, 8H, H-5/6/7), 3.91 – 3.84 (m, 4H, H-17) 3.85 (s, 8H, H-15), 3.76 – 3.58 (m, 4H, H-5/6/7), 1.67 – 1.13 (m, 20H, H-18-23), 1.01 – 0.88 (m, 18H, H-24,25).

**^13^C NMR** (100.63 MHz, d-Aceton, 300K): δ[ppm] = 157.3 (C_q_, C-8/16), 155.8 (C_q_, C-8/16), 148.2 (C_q_, C-3), 135.6 (C_q_, C-9/14), 133.7 (C_q_, C-9/14), 131.0 (CH, C-10/13), 130.2 (CH, C-10/13), 123.0 (CH, C-1/11/12), 122.4 (CH, C-1/11/12), 121.5 (CH, C-1/11/12), 112.5 (CH, C-2), 72.8 (CH_2_, C-5/6/7), 70.0 (CH_2_, C-5/6/7), 69.5 (CH_2_, C-17), 69.1 (CH_2_, C-5/6/7), 67.3 (CH_2_, C-4), 39.2 (CH_2_, C-18/19/20/21/22), 37.7 (CH_2_, C-18/19/20/21/22), 36.8 (CH2, C-15), 35.8 (CH_2_, C-18/19/20/21/22), 30.3 (CH_2_, C-18/19/20/21/22), 27.9 (CH_2_, C-23), 24.8 (CH_2_, C-18/19/20/21/22), 22.2 (CH3, C-25), 19.7 (CH3, C-24).

**^19^F NMR** (376.50 MHz, d-Aceton, 300K): δ[ppm] = -78.78 (CF_3_SO_3_^-^).

**MS (ESI^+^):** [M^+^] = NH_4_C_62_H_82_O_8_, calculated: 972.6353, found: 972.6347.

### [Na(MAXCalix)]OTf


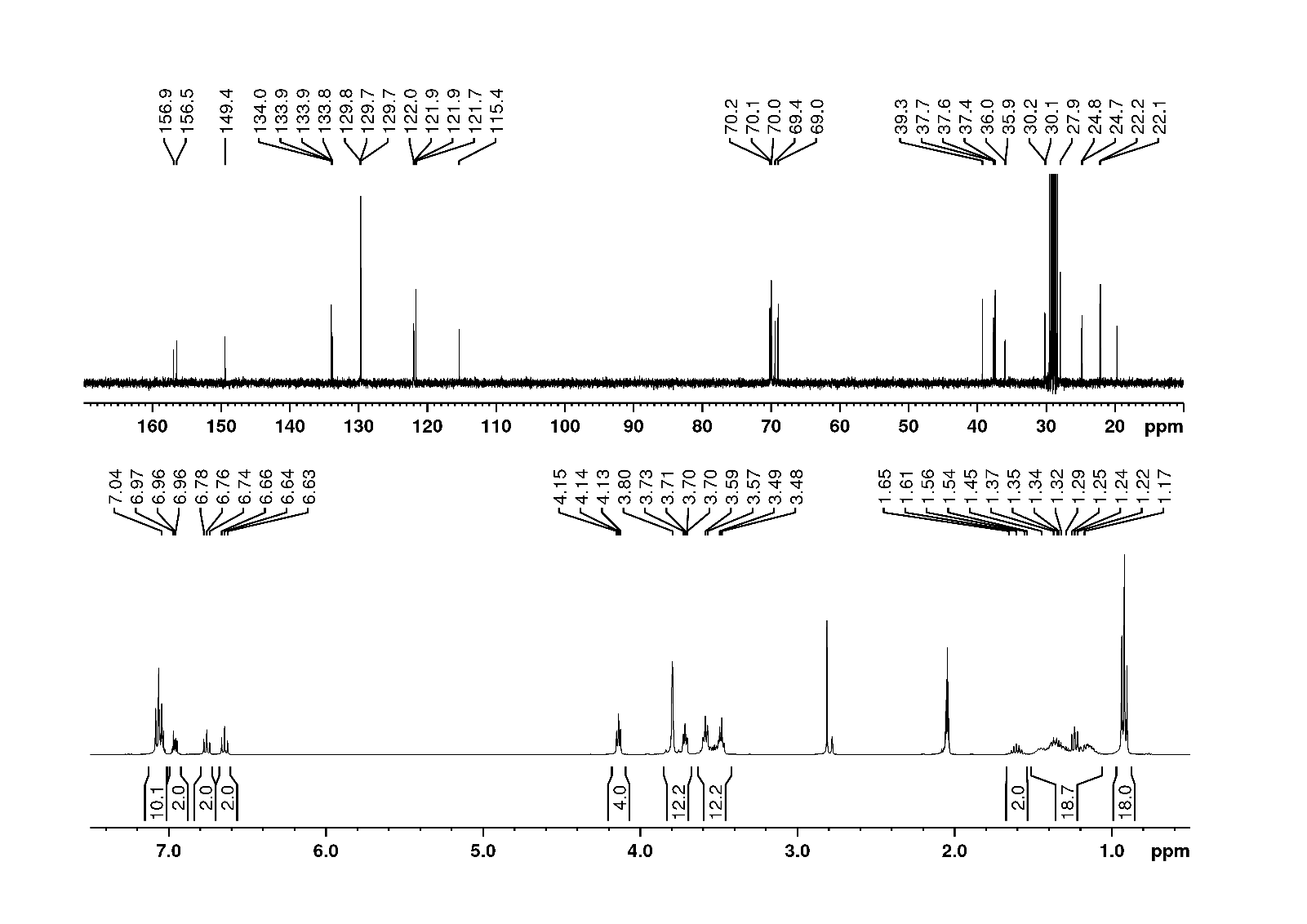


**Figure S13.** ^1^H (bottom, 400.17 MHz, 300K) and ^13^C (top, 100.63 MHz, 300K) spectrum of [Na(MAXCalix)]OTf in acetone-d_6_.


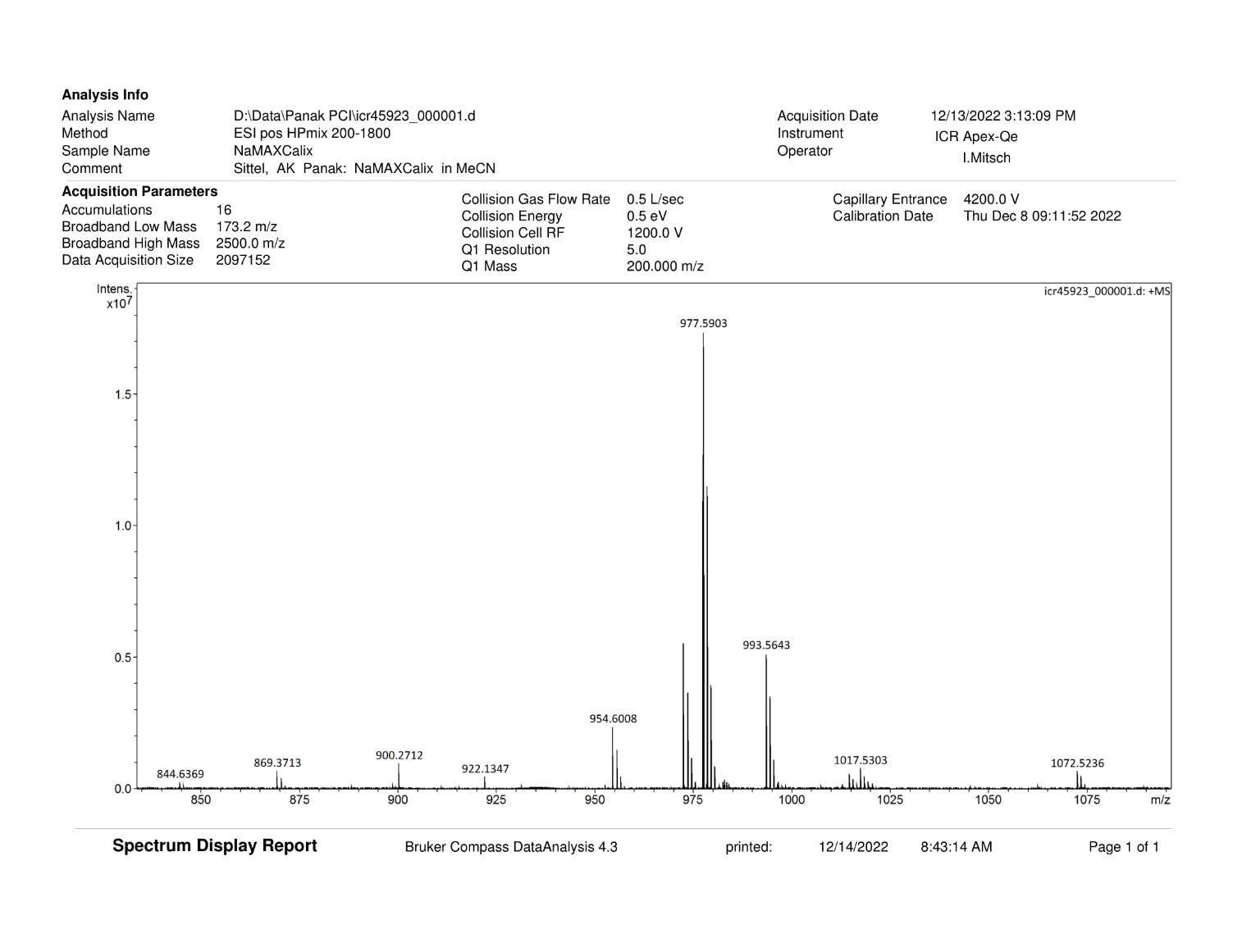


**Figure S14.** ESI^+^ mass spectrum of [Na(MAXCalix)]^+^.

**^1^H NMR** (400.17 MHz, d-Aceton, 300K): δ[ppm] = 7.11 – 7.02 (m, 10H, H-2,10,13), 6.99 – 6.93 (m, 2H, H-1), 6.76 (t, *J*=7.5, 2H, H-11), 6.64 (t, *J*=7.5, 2H, H-12), 4.14 (t, *J*=5.0, 4H, H-4-7,17), 3.80 (s, 8H, H-15), 3.71 (td, *J*=5.0, 1.4, 4H, H-4-7,17), 3.63 – 3.44 (m, 12H, H-4-7,17), 1.68 – 1.53 (m, 2H, H-23), 1.51 – 1.08 (m, 18H, H-18-22), 1.00 – 0.86 (m, 18H, H-24,25).

**^13^C NMR** (100.63 MHz, d-Aceton, 300K): δ[ppm] = 156.9 (C_q_, C-8/16), 156.5 (C_q_, C-8/16), 149.4 (C_q_, C-3), 134.0 (C_q_, C-9/14), 133.9 (C_q_, C-9/14), 129.8 (CH, C-10/13), 129.8 (CH, C-10/13), 122.0 (CH, C-1/11/12), 121.9 (CH, C-1/11/12), 121.7 (CH, C-1/11/12), 115.4 (CH, C-2), 70.2 (CH_2_, C-4/5/6/7/17), 70.1 (CH_2_, C-4/5/6/7/17), 70.0 (CH_2_, C-4/5/6/7/17), 69.4 (CH_2_, C-4/5/6/7/17), 69.0 (CH_2_, C-4/5/6/7/17), 39.3 (CH_2_, C-18/19/20/21/22), 37.7 (CH_2_, C-18/19/20/21/22), 37.4 (CH2, C-15), 36.0 (CH_2_, C-18/19/20/21/22), 30.2 (CH_2_, C-18/19/20/21/22), 27.9 (CH_2_, C-23), 24.8 (CH_2_, C-18/19/20/21/22), 22.2 (CH3, C-25), 19.7 (CH_3_, C-24).

**^19^F NMR** (376.50 MHz, d-Aceton, 300K): δ[ppm] = -78.94 (CF_3_SO_3_^-^).

**MS (ESI^+^):** [M^+^] = NaC_62_H_82_O_8_, calculated: 977.5907, found: 977.5903.

### [Sr(MAXCalix)]OTf_2_


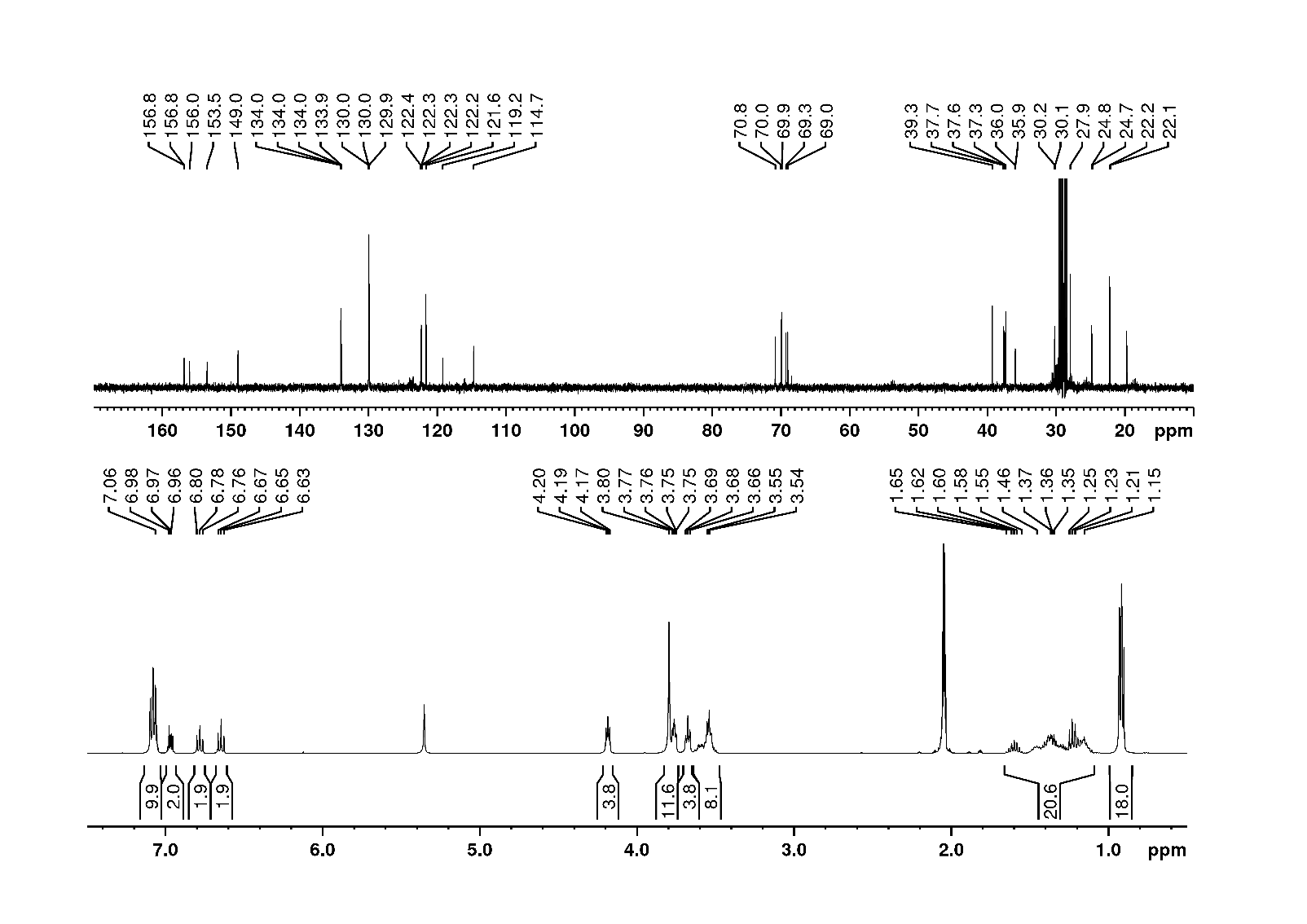


**Figure S15.** ^1^H (bottom, 400.17 MHz, 300K) and ^13^C (top, 100.63 MHz, 300K) spectrum of [Sr(MAXCalix)]OTf_2_ in acetone-d_6_.


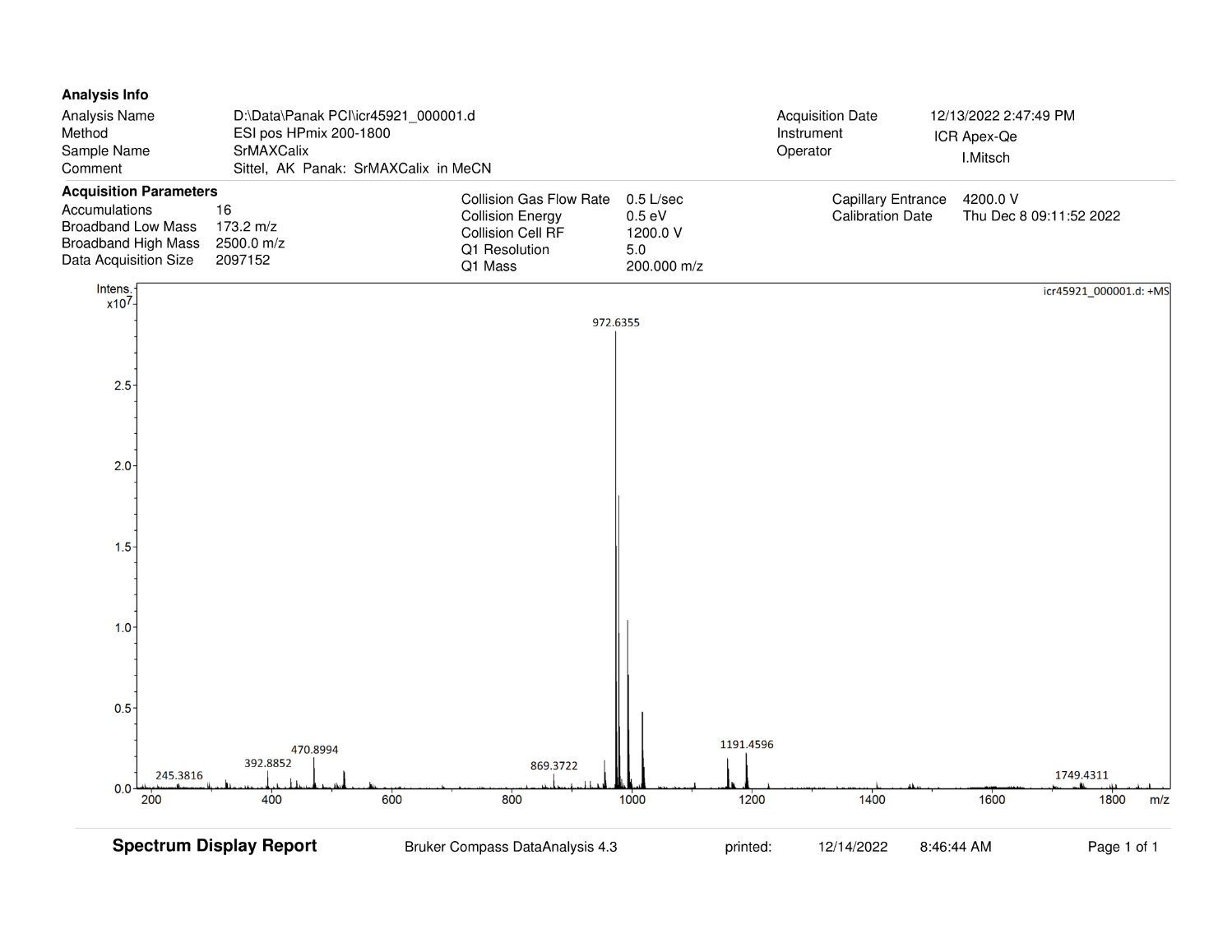


**Figure S16.** ESI^+^ mass spectrum of [Sr(MAXCalix)]OTf^+^.

**^1^H NMR** (400.17 MHz, d-Aceton, 300K): δ[ppm] = 7.13 – 7.03 (m, 10H, H-2,10,13), 7.00 – 6.93 (m, 2H, H-1), 6.78 (t, *J*=7.5, 2H, H-11), 6.65 (t, *J*=7.5, 2H, H-12), 4.19 (t, *J*=5.0, 4H, H-4-7,17), 3.80 (s, 8H, H-15), 3.78 – 3.74 (m, 4H, H-4-7/17), 3.68 (t, *J*=5.5, 4H, H-4-7,17), 3.64 – 3.49 (m, 8H, H-4-7,17), 1.66 – 1.54 (m, 2H, H-23), 1.51 – 1.09 (m, 18H, H-18-22), 0.97 – 0.87 (m, 18H, H-24,25).

**^13^C NMR** (100.63 MHz, d-Aceton, 300K): δ[ppm] = 156.8 (C_q_, C-8/16), 156.5 (C_q_, C-8/16), 149.0 (C_q_, C-3), 134.0 (C_q_, C-9/14), 134.0 (C_q_, C-9/14), 130.0 (CH, C-10/13), 130.0 (CH, C-10/13), 122.3 (CH, C-1/11/12), 121.6 (CH, C-1/11/12), 119.2 (CH, C-1/11/12), 114.7 (CH, C-2), 70.8 (CH_2_, C-4/5/6/7/17), 70.0 (CH_2_, C-4/5/6/7/17), 69.9 (CH_2_, C-4/5/6/7/17), 69.3 (CH_2_, C-4/5/6/7/17), 69.0 (CH_2_, C-4/5/6/7/17), 39.3 (CH_2_, C-18/19/20/21/22), 37.7 (CH_2_, C-18/19/20/21/22),37.3 (CH2, C-15), 35.9 (CH_2_, C-18/19/20/21/22), 30.2 (CH_2_, C-18/19/20/21/22), 27.9 (CH_2_, C-23), 24.8 (CH_2_, C-18/19/20/21/22), 22.2 (CH_3_, C-25), 19.7 (CH_3_, C-24).

**^19^F NMR** (376.50 MHz, d-Aceton, 300K): δ[ppm] = -79.16 (CF_3_SO_3_^-^).

**MS (ESI^+^):** [M^+^] = SrC_62_H_82_O_8_CF_3_SO_3_, calculated: 1191.4593, found: 1191.4596.

## Competitive Speciation Study


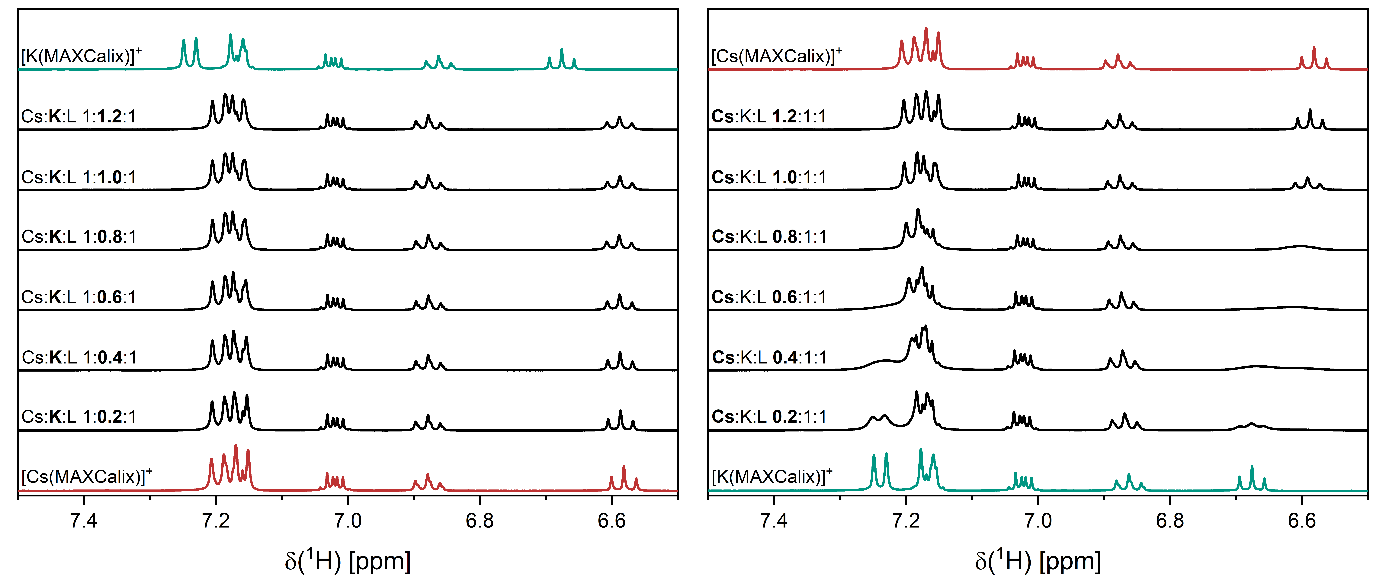

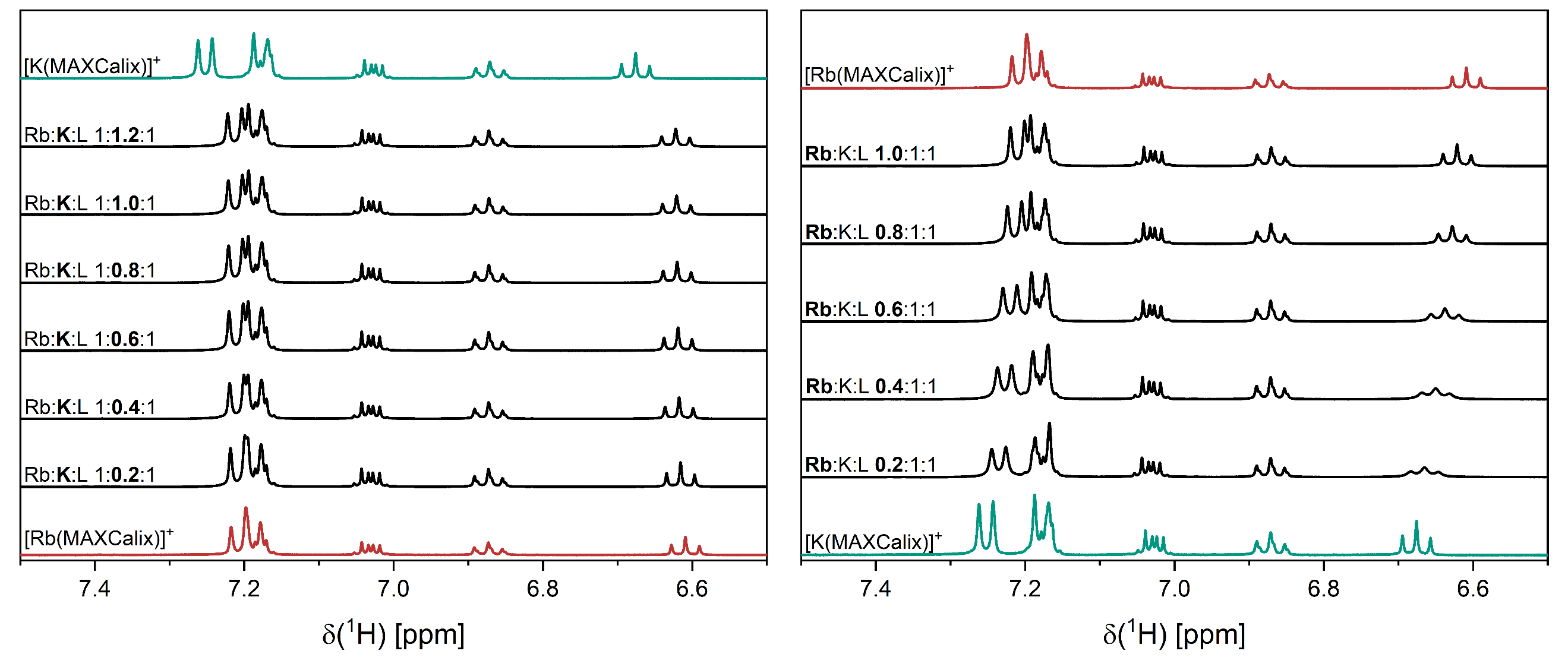

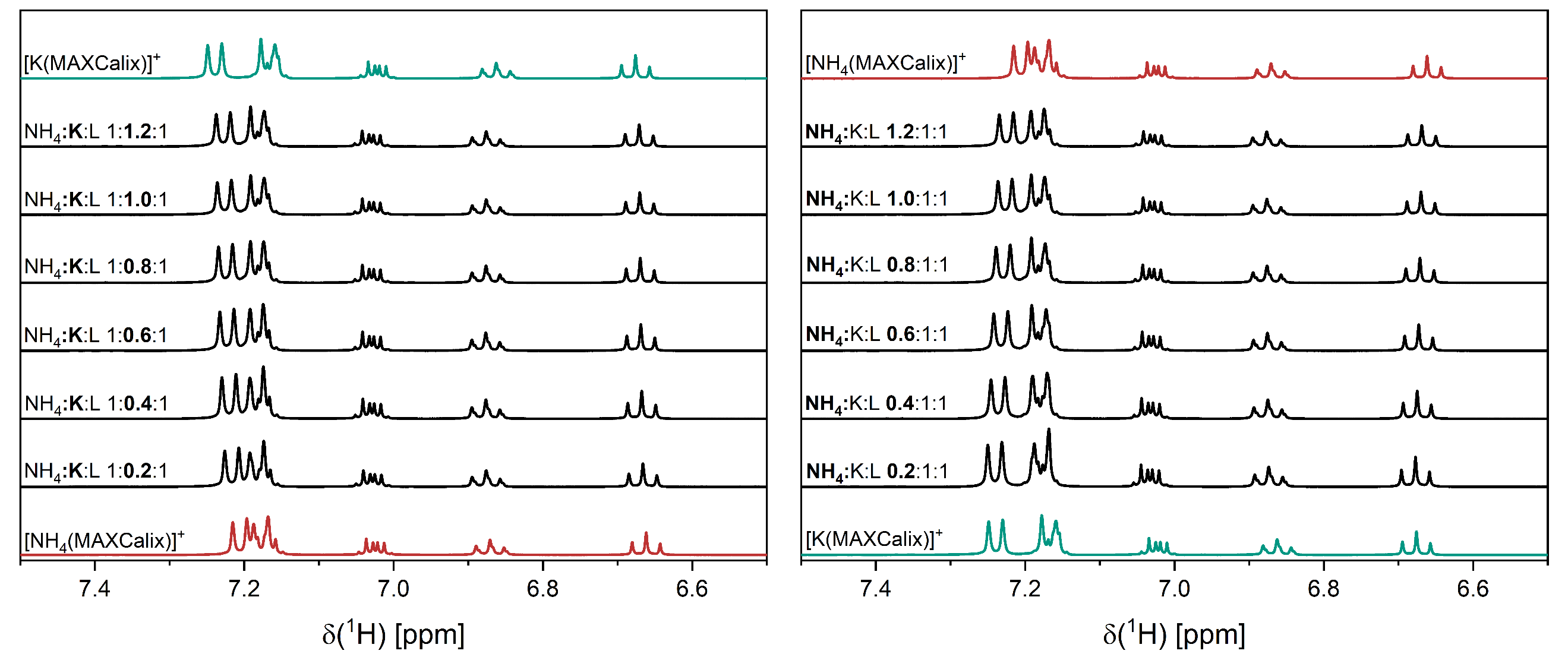


**Figure S17.** Evolution of the ^1^H-NMR spectra (400.13 MHz, 300 K) of the aromatic region of MAXCalix depending on the MAXCalix/metal ion ratio for the ion pairs Cs^+^/K^+^, Rb^+^/K^+^, NH_4_^+^/K^+^ ([M] = 1.7∙10^-2^ mol L^-1^) in acetone-d_6_.


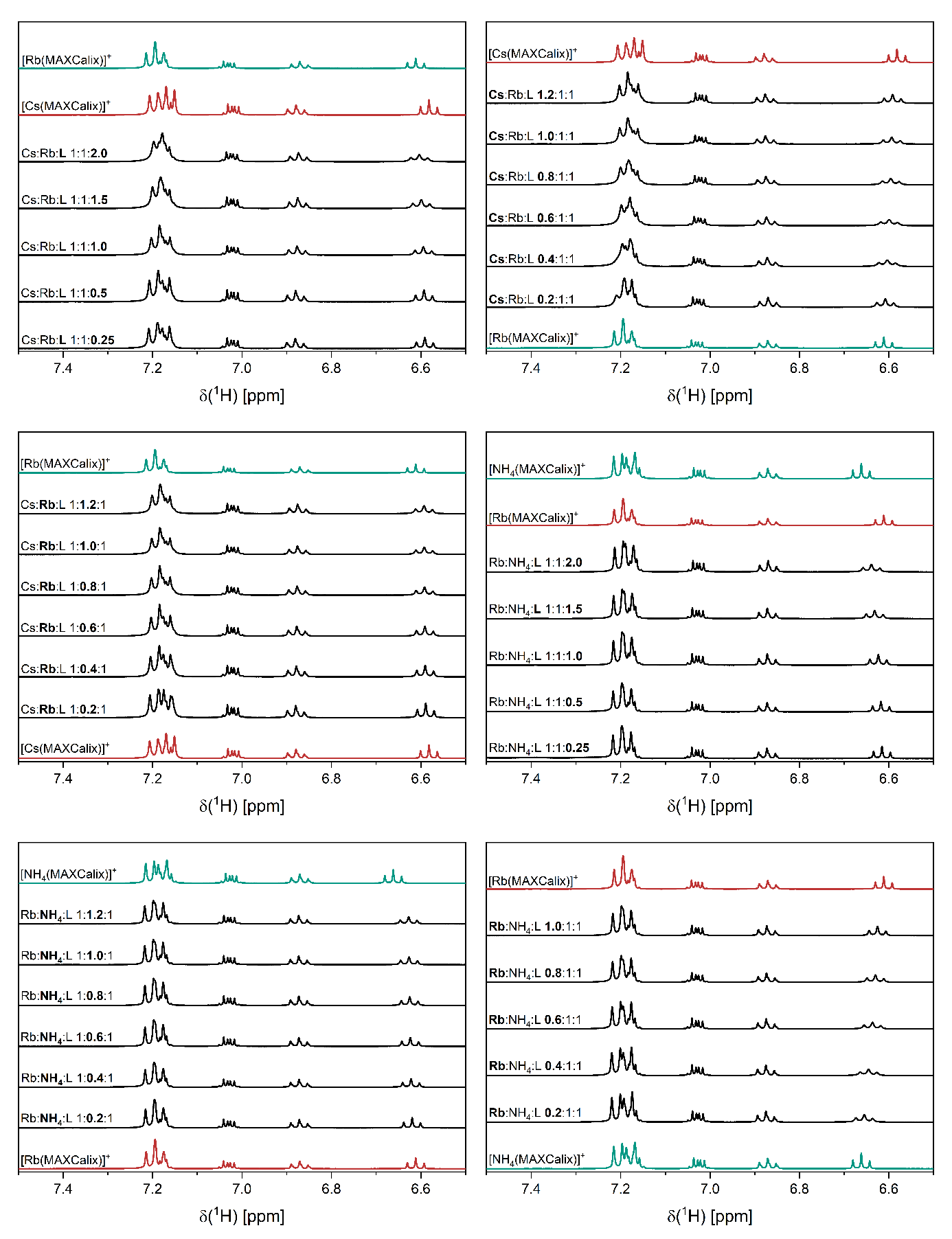


**Figure S18.** Evolution of the ^1^H-NMR spectra (400.13 MHz, 300 K) of the aromatic region of MAXCalix depending on the MAXCalix/metal ion ratio for the ion pairs Cs^+^/Rb^+^, Rb^+^/NH_4_^+^ ([M] = 1.7∙10^-2^ mol L^-1^) in acetone-d_6_.

## DFT structures


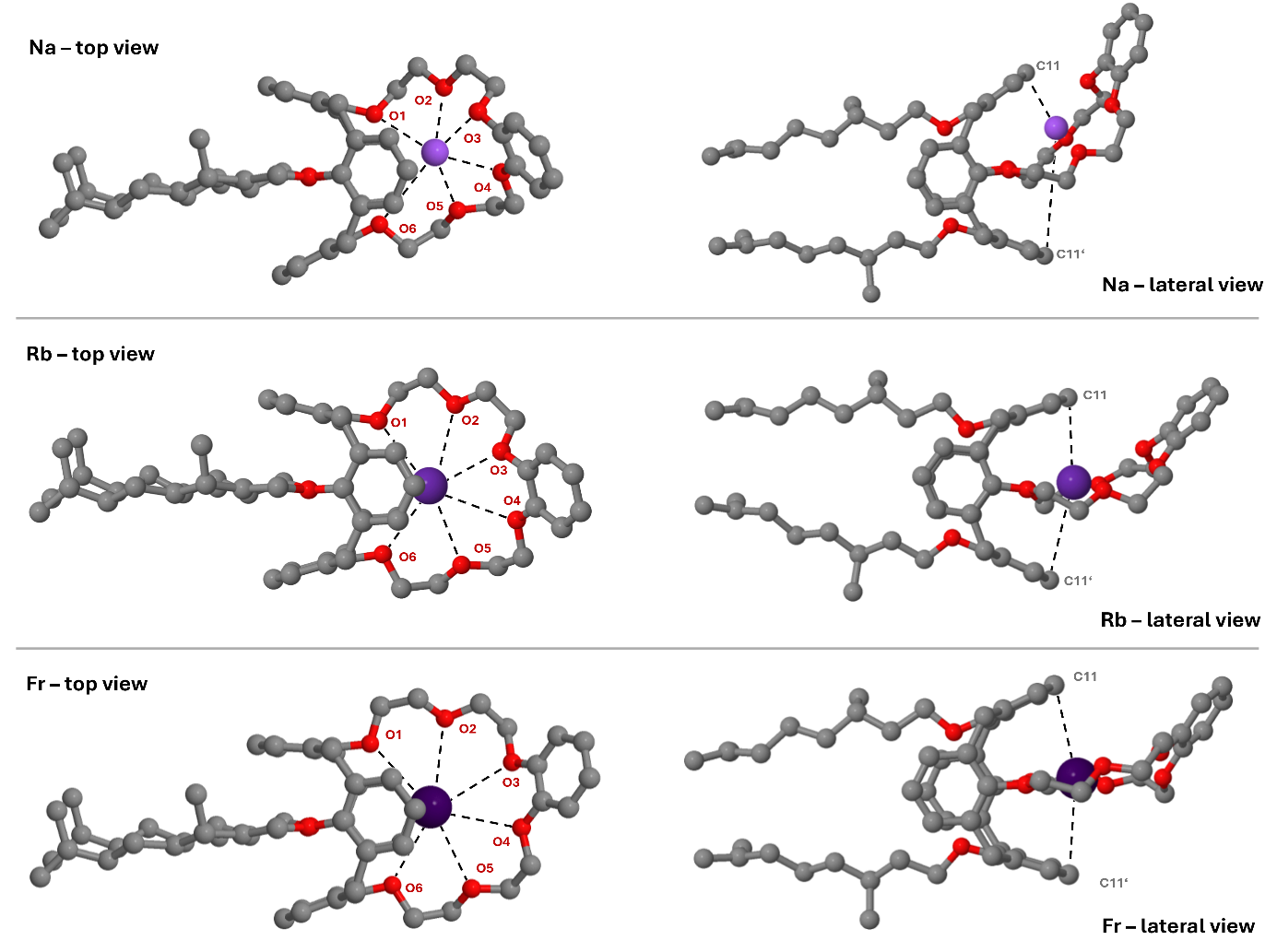


**Figure S19.** Top and lateral view of the DFT-optimized structures of [M(MAXCalix)]^+^ complexes (M = Na^+^, Rb^+^, Fr^+^) using BP86 functional with the def2-TZVPP basis set for Na^+^ and Rb^+^ and def-TZVP basis set for Fr^+^. Hydrogen atoms are omitted for visual clarity.


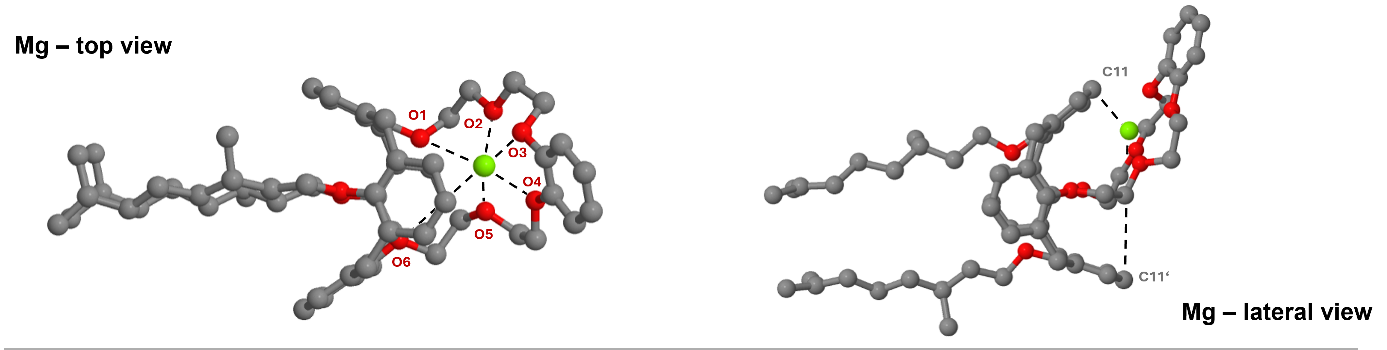

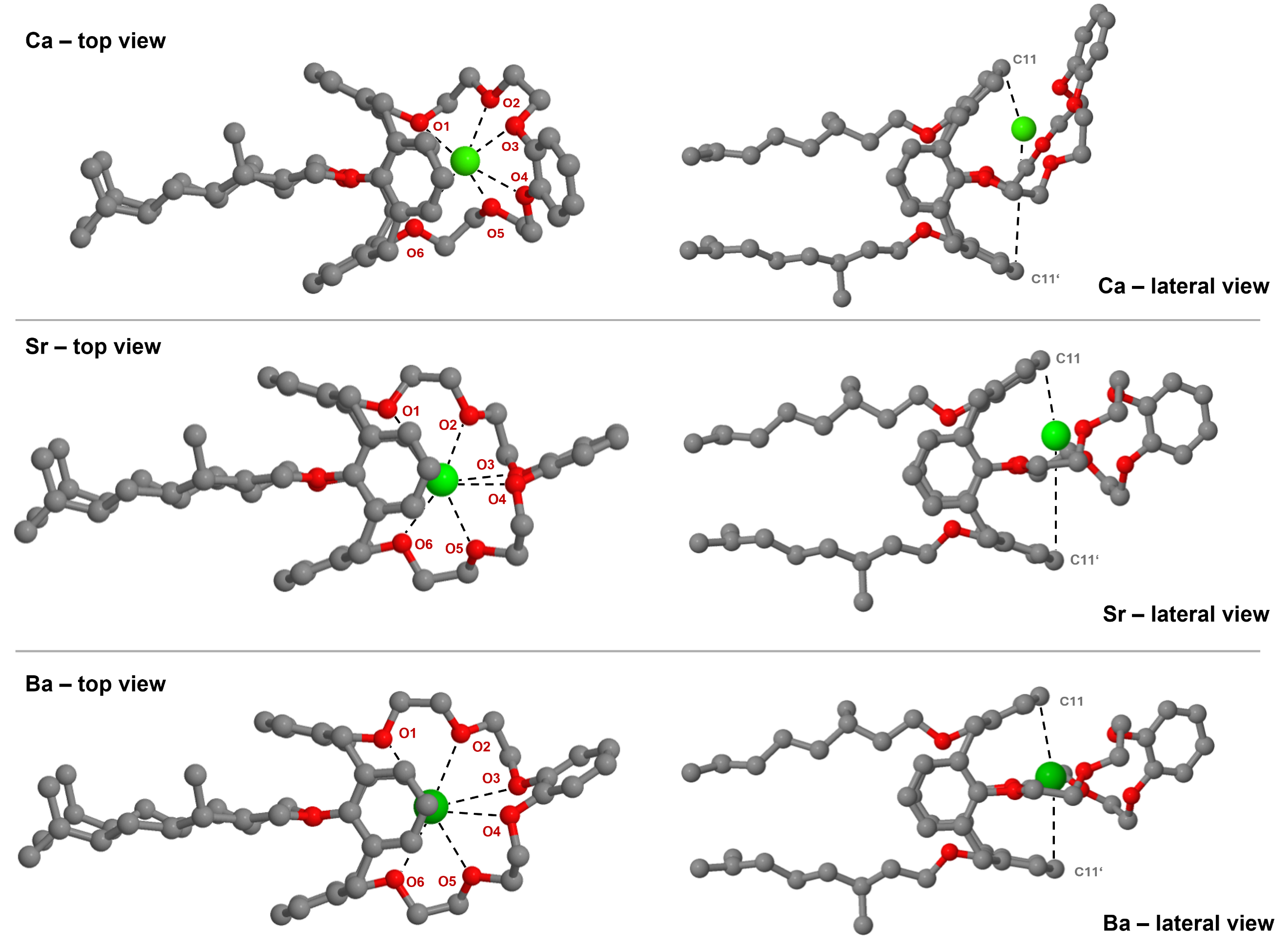


**Figure S20.** Top and lateral view of the DFT-optimized structures of [M(MAXCalix)]^2+^ complexes (M = Mg^2+^, Ca^2+^, Sr^2+^, Ba^2+^) using BP86 functional with the def2-TZVPP basis set. Hydrogen atoms are omitted for visual clarity.

## References

(1) Hohenberg, P.; Kohn, W. Inhomogeneous Electron Gas. *Physical Review* **1964**, *136* (3B), B864-B871. DOI: 10.1103/PhysRev.136.B864.

(2) Kohn, W.; Sham, L. J. Self-Consistent Equations Including Exchange and Correlation Effects. *Physical Review* **1965**, *140* (4A), A1133-A1138. DOI: 10.1103/PhysRev.140.A1133.

(3) Ahlrichs, R.; Furche, F.; Grimme, S. Comment on “Assessment of exchange correlation functionals” [A.J. Cohen, N.C. Handy, Chem. Phys. Lett. 316 (2000) 160–166]. *Chemical Physics Letters* **2000**, *325* (1), 317-321. DOI: <https://doi.org/10.1016/S0009-2614(00)00654-0>.

(4) Weigend, F.; Ahlrichs, R. Balanced basis sets of split valence, triple zeta valence and quadruple zeta valence quality for H to Rn: Design and assessment of accuracy. *Phys. Chem. Chem. Phys.* **2005**, *7* (18), 3297-3305, 10.1039/B508541A. DOI: 10.1039/B508541A.

(5) Weigend, F.; Häser, M.; Patzelt, H.; Ahlrichs, R. RI-MP2: optimized auxiliary basis sets and demonstration of efficiency. *Chemical Physics Letters* **1998**, *294* (1), 143-152. DOI: <https://doi.org/10.1016/S0009-2614(98)00862-8>.

(6) Eichkorn, K.; Weigend, F.; Treutler, O.; Ahlrichs, R. Auxiliary basis sets for main row atoms and transition metals and their use to approximate Coulomb potentials. *Theoretical Chemistry Accounts* **1997**, *97* (1), 119-124. DOI: 10.1007/s002140050244.

(7) Deglmann, P.; May, K.; Furche, F.; Ahlrichs, R. Nuclear second analytical derivative calculations using auxiliary basis set expansions. *Chemical Physics Letters* **2004**, *384* (1), 103-107. DOI: <https://doi.org/10.1016/j.cplett.2003.11.080>.

(8) Ahlrichs, R.; Furche, F.; Hättig, C.; Klopper, W. M.; Sierka, M.; Weigend, F. *TURBOMOLE v7.0, 2015*.

(9) Eichkorn, K.; Treutler, O.; Öhm, H.; Häser, M.; Ahlrichs, R. Auxiliary basis sets to approximate Coulomb potentials. *Chemical Physics Letters* **1995**, *240* (4), 283-290. DOI: <https://doi.org/10.1016/0009-2614(95)00621-A>.

(10) Schäfer, A.; Horn, H.; Ahlrichs, R. Fully optimized contracted Gaussian basis sets for atoms Li to Kr. *The Journal of Chemical Physics* **1992**, *97* (4), 2571-2577. DOI: 10.1063/1.463096 (acccessed 9/24/2024).

(11) Treutler, O.; Ahlrichs, R. Efficient molecular numerical integration schemes. *The Journal of Chemical Physics* **1995**, *102* (1), 346-354. DOI: 10.1063/1.469408 (acccessed 9/24/2024).

(12) von Arnim, M.; Ahlrichs, R. Geometry optimization in generalized natural internal coordinates. *The Journal of Chemical Physics* **1999**, *111* (20), 9183-9190. DOI: 10.1063/1.479510 (acccessed 9/24/2024).

(13) Tasi, A.; Gaona, X.; Fellhauer, D.; Böttle, M.; Rothe, J.; Dardenne, K.; Polly, R.; Grivé, M.; Colàs, E.; Bruno, J.; et al. Thermodynamic description of the plutonium – α-d-isosaccharinic acid system I: Solubility, complexation and redox behavior. *Applied Geochemistry* **2018**, *98*, 247-264. DOI: <https://doi.org/10.1016/j.apgeochem.2018.04.014>.

(14) Tasi, A.; Gaona, X.; Fellhauer, D.; Böttle, M.; Rothe, J.; Dardenne, K.; Polly, R.; Grivé, M.; Colàs, E.; Bruno, J.; et al. Thermodynamic description of the plutonium – α–d–isosaccharinic acid system ii: Formation of quaternary Ca(II)–Pu(IV)–OH–ISA complexes. *Applied Geochemistry* **2018**, *98*, 351-366. DOI: <https://doi.org/10.1016/j.apgeochem.2018.06.014>.

(15) Szabo, P. G.; Tasi, A. G.; Gaona, X.; Polly, R.; Maier, A. C.; Hedström, S.; Altmaier, M.; Geckeis, H. Solubility of Ca(ii), Ni(ii), Nd(iii) and Pu(iv) in the presence of proxy ligands for the degradation of polyacrylonitrile in cementitious systems. *Dalton Transactions* **2022**, *51* (24), 9432-9444, 10.1039/D2DT01409B. DOI: 10.1039/D2DT01409B.

(16) Shang, C.; Gaona, X.; Oher, H.; Polly, R.; Skerencak-Frech, A.; Duckworth, S.; Altmaier, M. Experimental and computational evidence of U(VI)-OH-Si(OH)(4) complexes under alkaline conditions: Implications for cement systems. *Chemosphere* **2024**, *350*, 141048. DOI: 10.1016/j.chemosphere.2023.141048 From NLM Medline.

(17) Comins, M. B.; Shang, C.; Polly, R.; Skerencak-Frech, A.; Altmaier, M.; Hixon, A. E.; Gaona, X. Cm(III) speciation in the presence of citrate from neutral to hyperalkaline conditions and the effect of calcium. *Chemosphere* **2024**, *364*, 143233. DOI: 10.1016/j.chemosphere.2024.143233 From NLM Medline.
